# Clonal hematopoiesis–derived therapy-related myeloid neoplasms after autologous hematopoietic stem cell transplant for lymphoid and non-lymphoid disorders

**DOI:** 10.1038/s41375-024-02258-y

**Published:** 2024-04-29

**Authors:** Hussein Awada, Carmelo Gurnari, Valeria Visconte, Arda Durmaz, Teodora Kuzmanovic, Hassan Awada, Zheng Jin Tu, James R. Cook, Brian J. Bolwell, Ronald Sobecks, Matt Kalaycio, David Bosler, Jaroslaw P. Maciejewski

**Affiliations:** 1 Translational Hematology and Oncology Research Department of Cleveland Clinic, Cleveland, NY USA; 2 Department of Biomedicine and Prevention, University of Rome Tor Vergata, Rome, Italy; 3 Roswell Park Comprehensive Cancer Center, Buffalo, NY USA; 4 Department of Laboratory Medicine, Cleveland Clinic, Cleveland, OH USA; 5 Department of Hematology and Oncology, Taussig Cancer Institute, Cleveland Clinic, Cleveland, OH USA

**Keywords:** Acute myeloid leukaemia, Myelodysplastic syndrome, Clinical genetics

## Abstract

Therapy-related myeloid neoplasms (tMN) are complications of cytotoxic therapies. Risk of tMN is high in recipients of autologous hematopoietic stem cell transplantation (aHSCT). Acquisition of genomic mutations represents a key pathogenic driver but the origins, timing and dynamics, particularly in the context of preexisting or emergent clonal hematopoiesis (CH), have not been sufficiently clarified. We studied a cohort of 1507 patients undergoing aHSCT and a cohort of 263 patients who developed tMN without aHSCT to determine clinico-molecular features unique to post-aHSCT tMN. We show that tMN occurs in up to 2.3% of patients at median of 2.6 years post-AHSCT. Age ≥ 60 years, male sex, radiotherapy, high treatment burden ( ≥ 3 lines of chemotherapy), and graft cellularity increased the risk of tMN. Time to evolution and overall survival were shorter in post-aHSCT tMN vs. other tMN, and the earlier group’s mutational pattern was enriched in *PPM1D* and *TP53* lesions. Preexisting CH increased the risk of adverse outcomes including post-aHSCT tMN. Particularly, antecedent lesions affecting *PPM1D* and *TP53* predicted tMN evolution post-transplant. Notably, CH-derived tMN had worse outcomes than non CH-derived tMN. As such, screening for CH before aHSCT may inform individual patients’ prognostic outcomes and influence their prospective treatment plans. Presented in part as an oral abstract at the 2022 American Society of Hematology Annual Meeting, New Orleans, LA, 2022.

## Introduction

Therapy-related myeloid neoplasms (tMN) may present as myelodysplastic syndrome (tMDS) or acute myeloid leukemia (tAML) [1–3] but constitute a distinct and genetically heterogeneous class of high-risk secondary myeloid disorders, which occur as a late complication of antecedent cytotoxic therapies for primary malignancies [4]. Using suitable controls, we have previously shown that cases of MN as second cancer coincidental or due to common genetic predisposition also fall into tMN and thereby contribute to the genetic diversity of this category [5]. Our group has further suggested that tMNs can be due to de novo acquisition of genomic or cytogenetic lesions or clonal evolution via a stage of clonal hematopoiesis (CH) [5]. Irrespective of these nuances, tMN typically exhibit poorer outcomes likely as a result of complex genomic architecture, including complex karyotypes, del(5q)/5-, del(7q)/7-, and *TP53* mutations [5–8]. The latest proposed 2022 World Health Organization (WHO) classification emphasizes the need to enhance prior definitions by reclassifying tMN as MN post-cytotoxic therapy (MN post-CT), while advocating the use of genetic qualifiers pertaining to MDS- or AML-specific cytogenetic or molecular terminology (*e.g*., AML with *KMT2A* rearrangement ‘post-CT’) [3]. The 2022 International Consensus Classification employs “therapy-related” as a diagnostic qualifier following a genetically-informed pathologic diagnosis [2]. These recommendations provide a new emphasis on the aforementioned cyto-genomic diversity of tMN.

Among patients at risk for tMN, autologous hematopoietic stem cell transplant (aHSCT) for lymphoma and myeloma has some distinctive features, including the potential impact of hematopoietic stem cell (HSC) dose (graft size), mobilization stress, chemotherapy prior to the high intensity conditioning regimens and factors related to re-expansion of grafted hematopoiesis [9–11]. In analogy to conventional tMN, post-aHSCT tMN may be related due to antecedent CH, accelerated progression of preexisting CH vs. CH-induced by HSC depletion and conditioning regimen. Consequently, tMN following aHSCT can be a product of CH or be due to de novo MN initiated by strong driver mutations. We have previously shown that analysis of founder lesions can point toward the origins of MN [12]. While CH has been described in post-aHSCT tMN [5, 12], the dynamics of CH evolution and its role as a mere marker vs. true initiator of subsequent tMN have not been explored. Specifically, in aHSCT, a multitude of other factors may contribute to the risk of tMN [13–17].

Here, we took advantage of a large number of patients undergoing aHSCT at our institution with available serial bone marrow samples along with detailed clinical annotations and long follow-up time to study the clinical dynamics and genomic architecture of tMN. Our aim was to identify the special features of tMN post-aHSCT and disentangle the intricacies of clonal trajectories related to the presence of CH prior to this procedure.

## Methods

### Study design and patients

We studied 1507 patients who underwent aHSCT at the Cleveland Clinic in the period spanning January 1, 2010 and February 1, 2022. Our cohort included patients with multiple myeloma (MM) (*n* = 790), non-Hodgkin lymphoma (NHL) (*n* = 519), Hodgkin lymphoma (HL) (*n* = 136), in addition to pediatric testicular germ cell tumor (*n* = 32), brain (*n* = 23), and bone tumors (*n* = 7). We further assembled a comparison cohort of tMN patients (*n* = 263) who did not have a history of aHSCT. We then reviewed electronic medical records to identify patients who developed tMN post-aHSCT and collected clinical parameters before admission and at tMN diagnosis. Finally, we case-matched all post-aHSCT tMN patients (*n* = 35) to a control group of aHSCT patients who did not develop tMN (*n* = 70) (according to variables in Supplementary Table S4) after sufficient follow-up time with the goal of designing a 1:2 case-control study comparing the genomic makeup of the two groups (Supplementary Tables S1 and S4**;** Supplementary Fig. S1).

### Genomic studies

A diagnostic NGS panel of 63 most commonly mutated genes in myeloid disorders (Supplementary Table S2) was used to detect CH on serial bone marrow specimens ahead of planned aHSCT and at tMN diagnosis. Samples were collected after obtaining informed consent in accordance with the regulations set forth by the Institutional Review Board of the Cleveland Clinic and the Declaration of Helsinki. Using molecular barcode technology, we detected variant allele frequency (VAF) cut-off of ≥ 2% for CH mutations. Our decision of choice for the arbitrary VAF cut off was influenced by the aim to maximize the generalizability of our study so that its results can be applied to patients encountered in daily clinical practice while maintaining the integrity of our sequenced samples. Thus, the 2% cut off was determined in accordance with cornerstone studies on CH and relevant clinical molecular pathology reports used in a real world clinical practice, while adhering to the requirements of the minimum average coverage at diagnostic loci [18, 19]. Further information on sequencing techniques are provided in the **Supplementary appendix**. All variants were evaluated using GnomAD and ClinVar information. Non-somatic lesions were consequently excluded. Founder mutations based on VAF were determined using previously described criteria (Supplementary Table S3) [5, 20, 21]. The frequency of CH mutations in healthy controls and patients with solid tumors was derived from previously published databases [22–24].

### Statistical analysis

Time to tMN diagnosis was calculated from first exposure to chemotherapy in both post-aHSCT and other tMN patients. Kaplan Meier tests were employed to estimate cumulative incidence and overall survival outcomes. Cox hazards proportions models were used to analyze the independent impact of baseline variables on incidence of tMN post-aHSCT. Multivariate logistic regressions were used for comparison of the characteristics of contrasting cohorts. Fisher test or Chi-square and unpaired *t*-tests were used for comparison of qualitative and quantitative variables, respectively.

All statistical tests were two-sided, and a *P*-value < 0.05 was considered statistically significant. Analysis and data visualization were generated using the R package (4.0.0 R Core Team, R Foundation for Statistical Computing, Vienna, Austria), Excel Microsoft Office 365 (Redmond, WA), and GraphPad Prism (8.4.0, San Diego, CA). Further details are provided in the Data Supplement file.

## Results

### Clinical features of post-aHSCT tMN

We first analyzed the clinical features of tMN diagnosis in a cohort of 1507 patients who underwent aHSCT. With a median follow-up of 4.4 years (IQR 2.3–7.5), 35 patients (2.3%) developed a tMN at a median time of 2.6 years (IQR 1.4–4.3) after transplant. Among them, 6 cases initially presented with tAML (17.1%) and 29 cases with tMDS (82.9%), of whom 7 (24.1%) progressed to tAML after a median of 1.6 months (IQR 0.6–4.9). Among tMN cases, 66% originally had NHL (4.4% of originally transplanted NHL), 26% MM (1.1% of originally transplanted MM), and 6% HL (1.5% of originally transplanted HL).

**Fig. 1:**
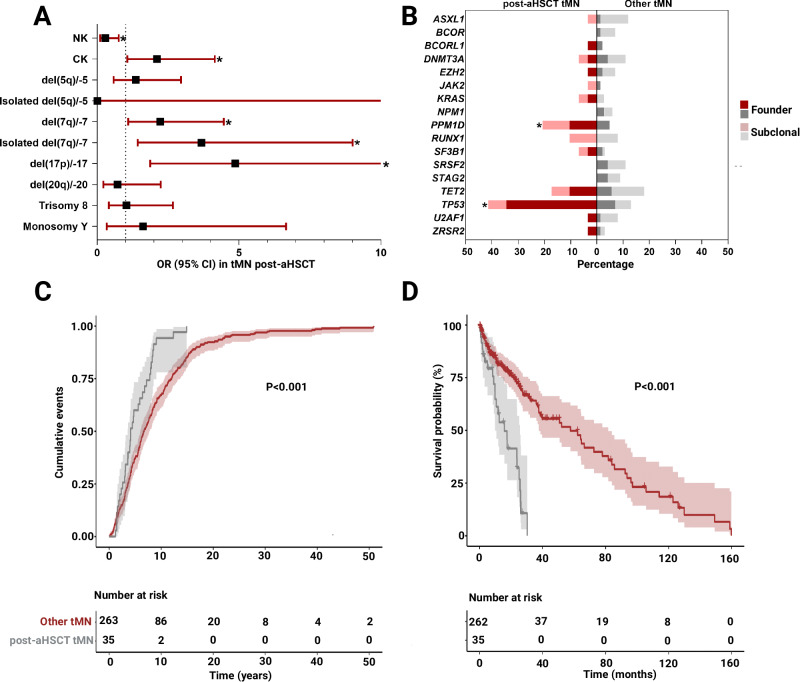
Characteristics of post-aHSCT tMN vs. other tMN. **A** Forest plot of Odds ratios (OR, and 95% CI) of cytogenetic abnormalities in post-aHSCT tMN relative to other tMN, with higher odds of post-aHSCT tMN to have complex karyotypes (OR 2.1, *P* = 0.5), del(7q)/7- (OR 2.2, *P* = 0.03), isolated del(7q)/7- (OR 3.7, *P* = 0.01) and del(17p)/17- (OR 4.7, *P* = 0.003), while other tMN patients are more likely to have normal karyotypes (OR 3.6, *P* = 0.02), with * denoting significance. **B** shows the mutational landscapes (top mutated genes) of post-aHSCT tMN compared to other tMN, including more frequent *PPM1D* (OR 5.1, *P* = 0.003) and *TP53* mutations (OR 4.9, *P* < 0·001) post-aHSCT, where * denotes *P* < 0.05. **C** Cumulative incidence demonstrates the significantly shorter latency period from first exposure to chemotherapy to tMN diagnosis in patients who had had subsequent aHSCT vs. no aHSCT (median 4.2 vs. 6.6 years, *P* < 0.001). **D** Kaplan-Meier curves showing the overall survival of post-aHSCT tMN to be significantly shorter than that of other tMN (median 17.7 vs. 57.7 months, *P* < 0.001).

When compared to our internal cohort of tMN following conventional chemotherapy (*n* = 263), post-aHSCT cases (*n* = 35) had similar age at both primary malignancy (median 62 vs. 60 years, *P* = 0.99) and tMN diagnosis (median 66.2 vs. 69, *P* = 0.38), but were more likely males (91 vs. 52%, *P* < 0.001; Table 1). tMDS post-aHSCT presented with a higher proportion of high-risk (HR) disease (determined by IPSS-R scores of ≥ 3.5; 79.3 vs. 43.2%, *P* = 0.03; Table 1), as substantiated by the increased frequencies of higher-risk chromosomal aberrations, including complex karyotypes (CK; 49 vs. 31%, *P* = 0.05), del(7q)/-7 (46 vs. 27%, *P* = 0.03) and del(17p)/-17 (23 vs. 6%, *P* = 0.003; Table 1 and Fig. 1A). On subsequent application of the *Molecular International Prognostic Score System (IPSS-M)*, a higher proportion of tMDS post-aHSCT was found to have very-high risk disease versus other tMDS (50% vs. 14.6%, *P* = 0.02) (Table 1).

**Table 1:** Demographic, clinical, and cytogenetic characteristics of post-aHSCT and other tMN.

Variables	All tMN	Post-aHSCT tMN	Other tMN	*P*-value
	*N* = 298	*N* = 35	*N* = 263	
**Demographics**				
**Age in years at first neoplasm, median (IQR)**	60.8 (51.9–67)	61.7 (55.4–65.2)	60.0 (51–68)	0.99
**Age in years at tMN, median (IQR)**	68.0 (60–75)	66·2 (58.4–69.5)	69.0 (61–76)	0.38
**Sex,** ***n*** **(%)**				<0.001
Male	168 (56.4)	32 (91.4)	136 (51.7)	
Female	130 (43.6)	3 (8.6)	127 (48.3)	
**tMN subtype,** ***n*** **(%)**				0.12
AML	84 (28.2)	6 (17.1)	78 (29.7)	
MDS	214 (71.8)	29 (82.9)	185 (70.3)	
**MDS risk category per IPSS-R,** ***n*** **(%)**				0.03
HR-MDS	103 (48.1)	23 (79.3)	80 (43.2)	
LR-MDS	111 (51.9)	6 (20.7)	105 (56.8)	
**MDS risk category per IPSS-M,** ***n*** **(%)**				0.02
Very low risk	6 (5.7)	1 (4.2)	5 (6.1)	
Low risk	24 (22.6)	3 (12.5)	21 (25.6)	
Moderately low risk	9 (8.5)	1 (4.2)	8 (9.8)	
Moderately high risk	16 (15.1)	3 (12.5)	13 (15.9)	
High risk	27 (25.5)	4 (16.6)	23 (28.0)	
Very high risk	24 (22.6)	12 (50)	12 (14.6)	
**Prior therapy**				
**Chemotherapy,** ***n*** **(%)**	209 (70.1)	35 (100)	174 (66.2)	<0.001
**Radiation therapy,** ***n*** **(%)**	190 (63.8)	13 (37.1)	177 (67.3)	<0.001
**Chemotherapy + radiation therapy,** ***n*** **(%)**	101 (33.9)	13 (37.1)	88 (33.5)	0.67
**Cytogenetics,** ***n*** **(%)**				
Normal	87 (29.2)	4 (11.4)	83 (31.6)	0.02
Abnormal	211 (70.8)	31 (88.6)	180 (68.4)	
Complex	98 (32.9)	17 (48.6)	81 (30.8)	0.05
**Karyotype abnormalities,** ***n*** **(%)**				
del(5q)/-5	62 (20.8)	9 (25.7)	53 (20.2)	0.51
Isolated del(5q)/-5	2 (0.7)	0 (0)	2 (0.8)	0.99
del(7q)/-7	88 (29.5)	16 (45.7)	72 (27.4)	0.03
Isolated del(7q)/-7	24 (8.1)	7 (20)	17 (6.5)	0.01
del(17p)/-17	23 (7.7)	8 (22.9)	15 (5.7)	0.003
Isolated del(17p)/-17	1 (0.3)	1 (2.9)	0 (0)	0.13
del(20q)	33 (11.1)	3 (8.6)	30 (11.4)	0.78
Isolated del(20q)	6 (2.0)	2 (5.7)	4 (1.5)	0.16
Trisomy (8)	42 (14.1)	5 (14.3)	37 (14.1)	0.99
Isolated Trisomy (8)	18 (6.0)	1 (2.9)	17 (6.5)	0.70
del(Y)	12 (4.0)	2 (5.7)	10 (3.8)	0.63
Isolated del(Y)	5 (1.7)	1 (2.9)	4 (1.5)	0.49

*aHSCT* autologous hematopoietic stem cell transplant, *tMN* therapy-related myeloid neoplasm, *IQR* interquartile range, *n* number, *%* percentage, *MDS* myelodysplastic syndrome, *AML* acute myeloid leukemia, *IPSS-R* Revised International Prognostic Scoring System, *IPSS-M* Molecular International Prognostic Scoring System, *LR* low risk, *HR* high risk according to Pfeilstöcker et al. Blood 2016, *del* deletion, (-) monosomy.

Somatic sequencing studies further revealed post-aHSCT tMN to be enriched with *PPM1D* (20.7 vs. 4.9%, *P* = 0.003) and *TP53* mutations (41.4 vs. 12.7%, *P* < 0.001), including multi-hit *TP53* allelic state (defined as ≥ 2 mutations, VAF > 30% or with -17/del17p; 31 vs. 11%, *P* = 0.02; Fig. 1B) compared to other tMN. The remainder of the genomic landscape appeared to be similar to that of tMN following other cytotoxic therapies (Supplementary Table S5). Differences in molecular and cytogenetic patterns were clinically paralleled by an earlier onset of tMN evolution post-aHSCT vs. no aHSCT (median 4.2 vs. 6.6 years, *P* < 0.001; Fig. 1C). tMN after aHSCT also resulted in a significantly shorter overall survival (median 18 vs. 58 months, *P* < 0.001; Fig. 1D) compared to tMN without aHSCT.

**Fig. 2:**
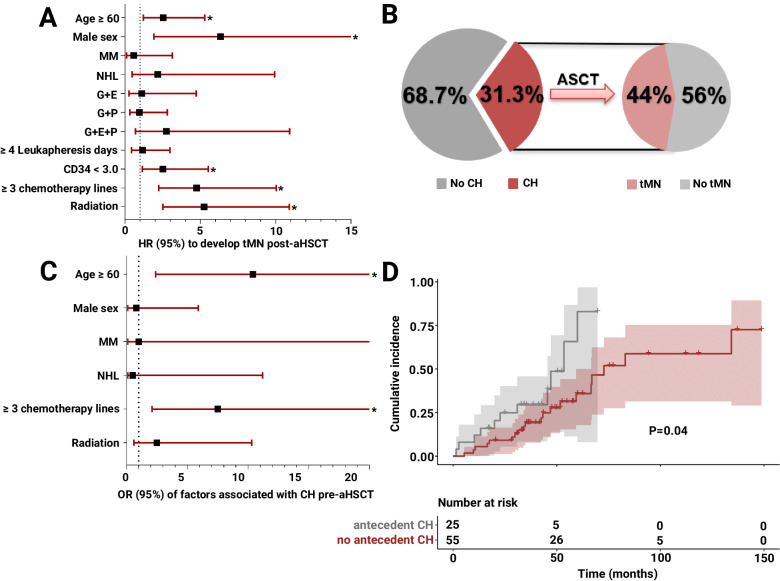
Risks of post-aHSCT tMN and study of CH prevalence pre-transplant. **A** Forest plot of the cox proportional hazard ratios of clinical factors that independently increase the risk of tMN post-aHSCT per multivariate analysis of 1507 patients undergoing aHSCT, including age ≥ 60 years at aHSCT (HR 2.5, 95% CI 1.2–5.3), male sex (HR 6.3, 95%CI 1.9–20.9), graft cellularity of CD34 + < 3.0 × 10^6^/Kg (HR 2.2, 95% CI 1.1–5.5), ≥ 3 lines of chemotherapy pre-aHSCT (HR 4.7, 95% CI 2.2–10.0), and prior radiation (OR 5.2, 95% CI 2.5–10.9), with * indicating *P* < 0·05. **B** illustrates the prevalence of CH pre-aHSCT in 31.3% of the patients, of whom 44% developed tMN post-aHSCT. **C** Forest plot of the OR of clinical factors that influence CH prevalence pre-aHSCT as per multivariate analysis of a case control cohort of 80 patients with available samples pre-aHSCT, including age ≥ 60 years (OR 10.4, 95% CI 2.4–64.2) and ≥ 3 lines of chemotherapy (OR 7.5, 95% CI 2.1–33.5), with * indicating statistical significance. **D** Cumulative incidence of tMN development post-aHSCT in patients with antecedent CH vs. others (median 53.5 vs. 72.8 months; *P* = 0.04).

When studying baseline clinical determinants of tMN development (Supplementary Table S6), our cox proportional hazards model showed that the risk of tMN is independently influenced by age ≥ 60 years at the time of aHSCT (HR 2.5, 95% CI 1.2–5.3), male sex (HR 6.3, 95% CI 1.9–20.9), graft cellularity (CD34+ dose < 3.0 × 10^6^/Kg; HR 2.5, 95% CI 1.5–5.5), high pre-aHSCT treatment burden ( ≥ 3 lines of chemotherapy; HR 4.7, 95% CI 2.2–10.0), and prior radiation (HR 5.2, 95% CI 2.5–10.9; Fig. 2A; Supplementary Table S7). Type of primary neoplasm, mobilization protocol, or number of leukapheresis procedures (Supplementary Table S7) did not increase risk of tMN.

### Clonal hematopoiesis is notably prevalent in patients undergoing aHSCT

Next, we sequenced pre-aHSCT samples of patients who did (*n* = 35) or did not develop tMN (*n* = 70) in a 1:2 ratio and equal follow-up times for comparison purposes. We identified a notable prevalence of CH in 31.3% (median VAF 5.2%, IQR 3.3–11.0) of our cohort. Out of these CH carriers, 44% developed tMN within the studied follow-up period (Fig. 2B).

**Fig. 3:**
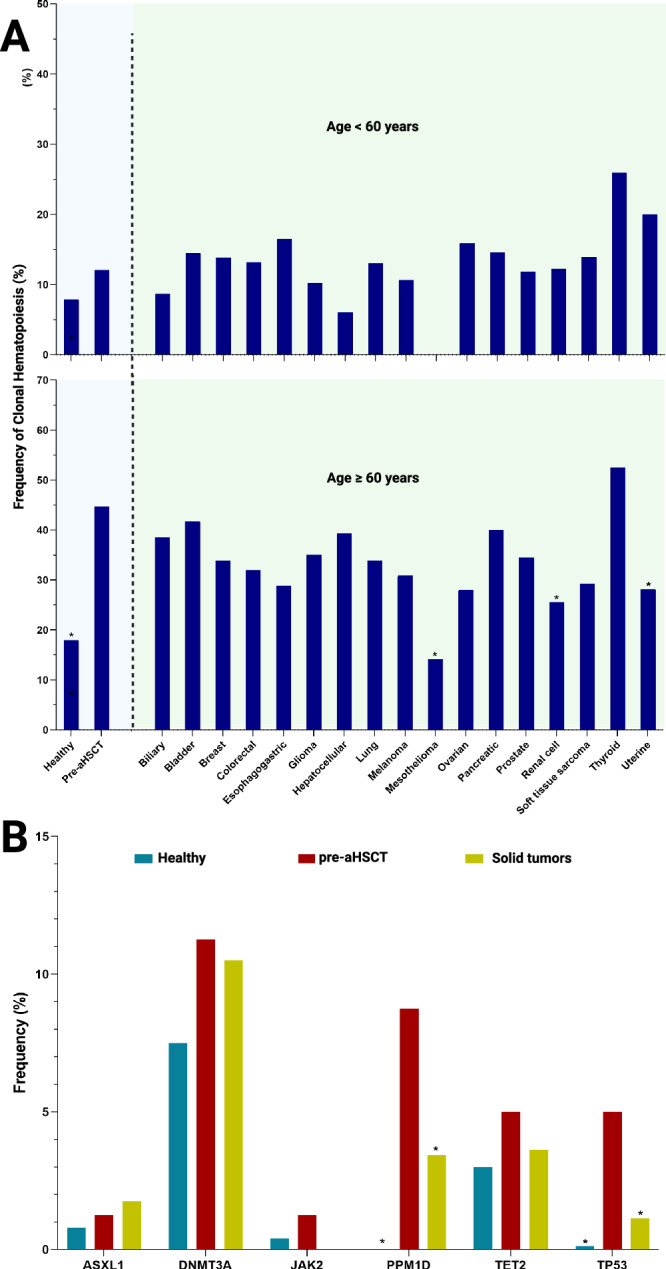
Prevalence of CH mutations pre-aHSCT compared to healthy patients and solid tumors. **A** Bar chart comparing the prevalence of CH in our pre-aHSCT cohort to healthy controls aged < 60 years (12.5 vs. 7.9%, *P* = 0.3) and ≥ 60 years (43.8 vs. 17.9%, *P* < 0.001), and to each of 17 types of solid tumors across the < 60 and ≥ 60 years age groups (Supplementary Fig. S8), with * indicating significant differences compared to the same age subgroup of our pre-aHSCT cohort. **B** Bar chart showing the higher frequency of lesions involving *PPM1D* (8.8 vs. 3.4%, *P* = 0.02) and *TP53* mutations (5.0 vs. 1.1%, *P* = 0.002) pre-aHSCT vs. all solid tumors.

Multivariate analysis identified age ≥ 60 years (OR 10.4, 95% CI 2.4–64.2) and ≥ 3 lines of chemotherapy (OR 7.5, 95% CI 2.1–33.5) as independent risk factors for CH pre-aHSCT (Fig. 2C; Supplementary Table S8). The presence of antecedent CH was further associated with worse outcomes post-aHSCT (median OS 42.7 vs. 72.8 months, *P* = 0.002) including faster rate of tMN evolution (median 53.5 vs. 72.8 months; *P* = 0.04, Fig. 2D). We then compared the frequency of CH in our cohort vs. age-matched controls using public databases of healthy individuals (*n* = 765) [23, 24]. The prevalence of CH in our cohort pre-transplant was higher than expected as opposed to controls when adjusted for age ( < 60 years = 12.5 vs. 7.9%, *P* = 0.3; ≥ 60 years = 43.8 vs. 17.9%, *P* < 0.001) [23, 24]. In contrast, CH pre-aHSCT had similar prevalence when compared to carriers of 17 solid tumor types (total *n* = 5649) for the same age categories ( < 60 years = 12.1 vs. 13.4%, *P* = 0.82; and ≥ 60 years = 44.4 vs. 33.4%, *P* = 0.11) (Fig. 3A; Supplementary Table S9) [22]. Particularly, patients undergoing aHSCT harbored more mutations in *PPM1D* (8.8 vs. 3.4%, *P* = 0.02) and *TP53* (5 vs. 1.1%, *P* = 0.002) but not in *ASXL1* (1.3 vs. 1.8%, *P* = 0.73), *TET2* (5 vs. 3.6%, *P* = 0.54) or *DNMT3A* (11.3 vs. 10.5, *P* = 0.83) as compared to patients with solid tumors (Fig. 3B; Supplementary Table S10) [22].

### CH-derived post-aHSCT tMN results from the transformation of an antecedent CH clone and has poor prognosis

**Fig. 4:**
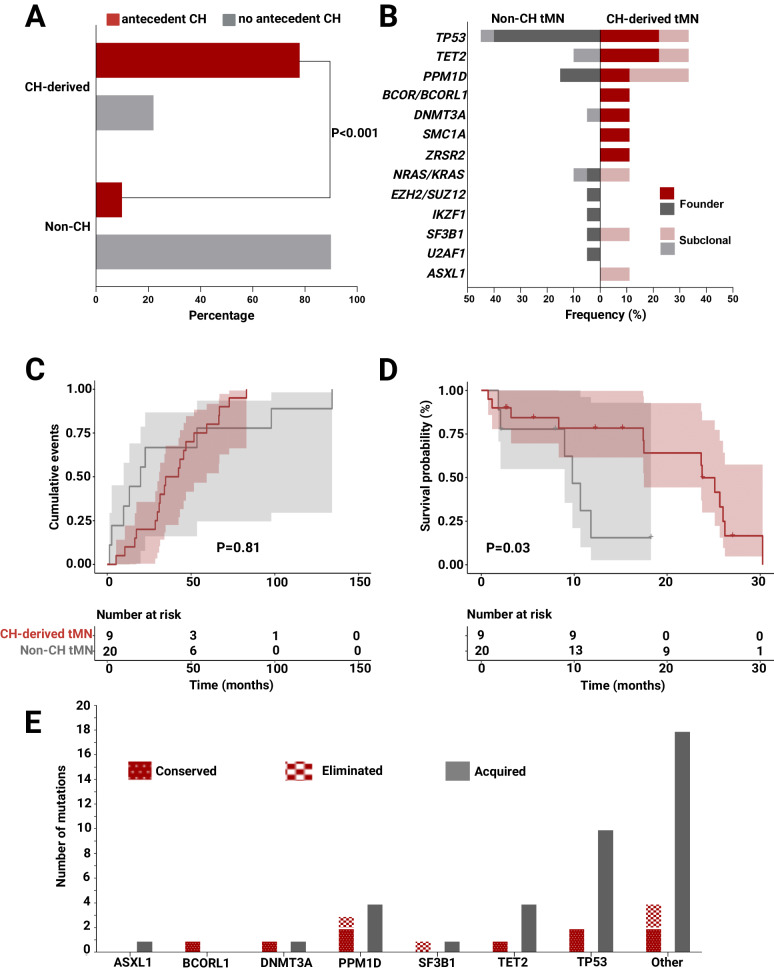
Characteristics of CH-derived vs. non-CH tMN post-aHSCT and clonal dynamics during aHSCT. **A** Bar chart showing the significantly higher OR of antecedent CH as a precursor to CH-derived tMN post-aHSCT (OR 31.5, *P* < 0.001). **B** compares the molecular landscape of CH-derived vs. non-CH tMN, with CH-derived disease being *TET2* and *TP53* predominant while non-CH tMN primarily *TP53*-related. **C** compares times to tMN diagnosis post-aHSCT in CH-derived vs. non-CH tMN (median 19.9 vs. 38.6 months, *P* = 0.81). In (**D**), Kaplan Meier curves demonstrate poorer prognosis of CH-derived tMN vs. non-CH disease post-transplant (median 9.8 vs. 23.8 months, *P* = 0.03), with disease aggressiveness noted by higher 1-year mortality rates (66.7 vs. 20%, *P* = 0.01). **E** Bar chart of gene-specific pre-aHSCT clones that were conserved, eliminated, and acquired through the transplant process as determined by comparisons between pre-aHSCT and post-aHSCT samples.

In previous studies, we have demonstrated that the presence of founder mutations typical of CH in evolved myeloid neoplasms may indicate derivation of these disease from CH [12]. The dissection of the genomic architecture allowed the identification of a CH-derived tMN in 9/29 (26%) cases, with 7/29 (20%) cases being related to antecedent CH, 2 derived from de novo CH evolving following transplant, while in 2/29 (5.7%) cases pre-aHSCT CH seemed to have disappeared following transplant (Supplementary Table S11). The majority of CH clones pre-aHSCT were conserved throughout the transplant process, as patients with CH-derived tMN were more likely to have detectable CH prior to the procedure (78 vs. 10%, OR 31.5, P < 0.001) than the non-CH tMN patients (Fig. 4A). Founder *TET2* (22.2%) and *TP53* (22.2%) mutations were the most common in CH-derived disease, followed by those in *PPM1D* (11.1%)*, BCOR/L1* (11.1%)*, DNTM3A* (11.1%)*, SMC1A* (11.1%) and *ZRSR2* (11.1%) genes (Fig. 4B). In contrast, tMN without antecedent CH clones were primarily related to dominant *TP53* mutations (40%), followed by lesions involving *PPM1D* (15%), *KRAS/NRAS* (5%), *EZH2/SUZ12* (5%), *SF3B1* (5%) and *U2AF1* (5%) (Fig. 4B). As such, CH-related tMN was primarily driven by *TET2* and *TP53* mutations, while non-CH tMN was mostly *TP53*-mediated. When we compared the pre- and post-aHSCT genomic architecture of the patients who developed tMN, around 76.5% of all clones (ancestral, subclonal or biallelic) were conserved throughout the post-transplant course, while 75.9% of patients acquired new mutations. Novel clones involved mostly *TP53* (25.6%), followed by *TET2* (10.3%) and *PPM1D* (10.3%; Fig. 4E). No significant differences were noted with regard to the time to tMN onset between CH-derived vs. non-CH cases (median 19.9 vs. 38.6 months; *P* = 0.81; Fig. 4C). However, CH-derived disease was more aggressive with significantly shorter survival (median 9.8 vs. 23.8 months; *P* = 0.03) and higher 1-year mortality rates (66.7 vs. 20%, *P* = 0.01; Fig. 4D**;** Supplementary Fig. S6).

### Ancestral *PPM1D* and *TP53* mutations pre-aHSCT may predict the development of tMN post-aHSCT

We subsequently explored the risk for tMN post-aHSCT in the setting of specific antecedent CH mutations within our follow-up period. After adjustment for demographic and clinical factors, our analysis suggested that pre-aHSCT cases with dominant or co-dominant mutations affecting *PPM1D* (12.9 vs. 2.0%, *P* = 0.05) and *TP53* (9.7 vs. 0%, P = 0.02) were more likely to be present in patients subsequently developing tMN (Supplementary Fig. S7). Besides, *PPM1D* or *TP53* mutation carriers showed a faster rate of tMN development (Supplementary Fig. S8). Similar differences were not detected for antecedent lesions involving *ASXL1* (0 vs. 2%, *P* = 0.42), *DNMT3A* (3.2 vs. 12.2%, *P* = 0.16), or *TET2* (3.2 vs. 6.1%, *P* = 0.56). In addition, the presence of >1 antecedent mutation was comparable across both groups (16.1 vs. 10.2%, *P* = 0.47).

## Discussion

Occurrence of tMN post-aHSCT is a devastating event that regrettably poses the poorest prognosis across the tMN spectrum. However, the true nature of tMN may not necessarily be related to cytotoxic exposure but instead second cancers that occur either coincidentally or due to pre-existing genetic predisposition as previously argued by our group [5]. However, a definitive reclassification cannot be possible without any erroneous degree and hence the latest WHO and ICC definitions still support the notion of therapy-relatedness in patients with prior cytotoxic exposure [2, 3].

In this study, we took advantage of detailed clinical annotation and availability of serial samples in a large cohort of patients who underwent aHSCT for various primary etiologies. Our approach includes identifying dominant and subclonal mutations according to previously published methods confirming the reliability of using a VAF difference of 5% in establishing clonal hierarchy [5, 20, 21, 25, 26]. We further define codominant mutations of VAF within 5% and merge them in analyses with dominant versus subclonal mutations. We show that the disproportionately short latency and poor survival of this cohort compared to other tMN is determined by high frequency of dismal features such as *TP53* lesions, del(7q)7- and CK. This finding correlated well with the increased frequency of higher-risk disease per both IPSS-R and IPSS-M scores despite the current lack of substantial evidence of the superiority of the latter in predicting tMDS outcomes [25, 27]. Nevertheless, we also demonstrate that the detection of CH is of clinical relevance since it further sub-stratifies post-aHSCT outcomes including tMN incidence and prognosis. The ability to follow clonal trajectories of patients who subsequently developed tMN along with respective case-matched non-progressors enabled several unique observations. Indeed, we revealed a high prevalence of CH (31.3%) in patients prior to aHSCT, with 44% of antecedent CH carriers developing tMN within the study follow-up period. We further demonstrate that the enhanced CH-related risk is primarily due to the transformation of the antecedent CH clones. The majority of antecedent CH-related tMN cases (85.7%) had significant clonal expansion post-transplant. In addition, we have confirmed that *TP53* and *PPM1D-*mutant CH constitute a risk factor for post-aHSCT tMN.

Overall, our results are in line with previously described smaller cohorts of aHSCT patients showing that CH may increase the risk of adverse outcomes including tMN post-aHSCT [28–31], though the effect of CH was not replicated in a study of 629 MM patients [28] and another of 420 lymphoma aHSCT cases [32]. Other studies have described limited subsets of patients with available NGS at tMN diagnosis [28, 29, 31, 33, 34]. In some of them, sequential sequencing in patients (*N* = 1 [29] ; *N* = 9 [31]; *N* = 10 [33]; *N* = 12 [34]; *N* = 13 [28]) post-aHSCT was suggested to evolve from acquisition of de novo mutations post-aHSCT or the transformation of pre-existing CH with or without clonal expansion [29, 31]. We have found a relatively similar prevalence of CH (31.3%) compared to previously reported percentages in the setting of aHSCT (43.1% [32]; 29.9% [31]; 25.5% [30]; 14.0% [28]) with analogous CH mutational pattern [28, 29, 31]. In our study, tMN was predominantly derived from *TP53* mutations, similar to the 42.9% [31] and 37.5% [33] reported in other studies. CH-derived tMN was also *TET2*-driven in 22.2% of our patients, as previously shown (28.6% [28], 18.2% [33] and 16.7% [31] of CH-derived CH). However, our report is the first to demonstrate the clinical significance of the molecular pathogenesis of CH-derived vs. non-CH tMN post-aHSCT, despite the predominant prevalence of *TP53* mutations in the latter.

In the general population, CH increases the relative risk for myeloid malignancy up to 13-fold [19, 35, 36] with the caveat of variable penetrance, and long latency period, thus resulting in an annual absolute risk for malignant transformation relatively low (estimated to be 0.5–1.0% per year) [37]. The prevalence of CH was significantly higher in our study population compared to what was reported in healthy individuals [23, 24]. While CH is known to increase with age, we further show its higher prevalence independent from age in patients with a high pre-treatment burden e.g., those who received ≥ 3 chemotherapy regimens, suggesting a cumulative effect. Consequently, we can estimate that the sub-cohort with antecedent CH has experienced an augmented annual risk of malignant transformation at 8.1–8.8% in the first two years following aHSCT. As such, one could stipulate that the amplified incidence of CH-related tMN is a consequence of an intrinsic effect of aHSCT, instead of a simple reflection of a higher prevalence of CH pre-aHSCT. Hence, we hypothesize a two-hit theory in which the conditioning stress imposed on the bone marrow and re-expansion of hematopoiesis (mimicking emergency hematopoiesis) after infusion of relatively low HSC numbers facilitate the selection of genetic facilitator hits of CH emergence (incurred pre-aHSCT) [38]. The latter scenario shares obvious mechanistic analogies with re-expansion of hematopoiesis after immunosuppressive treatment in aplastic anemia (e.g., bottleneck effect) and selection of CH seeds driving clonal evolution, as we recently showed [39]. Some other studies suggested that leukemia-permissive effects of conditioning and aHSCT confer selective pressure on non-infused surviving CH-clones in the underpopulated bone marrow [38]. A similar mechanism was proposed for the evolution of *TP53*-related CH pre-aHSCT [8]. In our cohort, 2 patients who had *TP53*-related CH experienced clonal expansion and subsequent tMN evolution without subclonal acquisition of new lesions. However, the majority of *TP53*-driven tMN were not related to antecedent CH.

Other factors affecting the acquisition and expansion of CH may be related to the specific types of drugs used, their duration, and effects on HSCs. Moreover, the mutational patterns resulting from such various effects may differ. For instance, CH in patients undergoing aHSCT shares common mutational predominance involving *DNMT3A* and *TET2* with other forms of CH but not *ASXL1* or *JAK2* [12, 35], which have been found in aging hematopoiesis. Similarly, our cohort exhibited enrichment in PPM1D and TP53 lesions which may possibly be explained by the more cytotoxic nature of MM and lymphoma regimens. Nevertheless, the pre-aHSCT mutational burden of TP53 and PPM1D (5% and 8.8%, respectively) was relatively comparable to that of non-aHSCT tMN at diagnosis (12.6% and 4.9%, respectively) suggesting that the subsequently enhanced TP53 and PPM1D clonal burden at diagnosis of post-aHSCT tMN may indeed be due to an intrinsic effect of the transplant process itself. Finally, antecedent CH was associated with a higher rate of adverse outcomes including tMN evolution. In addition, serial sampling asserted that CH-derived tMN was likely, but not exclusively, driven by antecedent CH. We further highlighted 3 other molecular mechanisms of post-aHSCT tMN, including non-antecedent CH-derived tMN, CH-eliminated tMN, and non-CH tMN (Supplementary Figs. S10 and S11). Whereas growth advantage of PPM1D mutant clones following HSCT was questioned [40], our results suggest that the presence of antecedent PPM1D may predispose to the development of tMN post-aHSCT. This notion is consistent with the impairment of recovery of normal hematopoiesis after transplant by PPM1D mutations or with their relative fitness advantage in particular when recovery is disturbed [41]. As such, screening for CH in all patients undergoing aHSCT evaluation may be warranted to highlight those at higher risk of clinical implications of CH-related tMN and adverse outcomes. This is especially pertinent in lymphoma and MM patients given the advent of alternative, perhaps less genotoxic options with lower tMN risk, such as bispecific antibodies and CAR T-cell therapy, as suggested in a recent study on patients with CLL developing tMN [42].

The main limitation of our study is its retrospective nature for which we dampen the conclusions that we derive from our results. Other limitations for our cohort include originating from a single center which may have restricted our sample size. Nevertheless, our paired samples are relatively larger than all other cohorts reported in th literature.

In conclusion, to the best of our knowledge, our study is the first to prove that the increased risk of post-aHSCT tMN triggered by CH is likely related to clonal selection and transformation of antecedent CH clones following the transplant process. We further demonstrated the dismal survival of post-aHSCT tMN in general, and the specifically poorer survival if the disease is CH-related. Our study provides a comprehensive revision of the clinical exposures that generally influence post-aHSCT tMN diagnosis in addition to induction-related CH in the modern era of lymphoid disorders treatments and aHSCT.

### Supplementary information

Supplemental material

## Data Availability

The authors have included relevant clinical and genetic data in the main text of the article and Supplementary Appendix. For additional information, please contact the corresponding author (maciejj@ccf.org).
