# Supplementary material for: Clonal hematopoiesis–derived therapy-related myeloid neoplasms after autologous hematopoietic stem cell transplant for lymphoid and non-lymphoid disorders
Source: Leukemia. 2024 Apr 29;38(6):1266–74. doi: 10.1038/s41375-024-02258-y (PMC11147764; doi:10.1038/s41375-024-02258-y)
Supplement: Supplementary file 1 — Supplemental material [file 41375_2024_2258_MOESM1_ESM.docx]

**Clonal hematopoiesis–derived therapy-related myeloid neoplasms after autologous hematopoietic stem cell transplant for lymphoid and non-lymphoid disorders**

**Hussein Awada^1#^ M.D., Carmelo Gurnari^1,2#^ M.D., Valeria Visconte^1^ Ph.D., Arda Durmaz^1^ Ph.D., Teodora Kuzmanovic^1^ B.A., Hassan Awada^3^ M.D., Zheng Jin Tu^4^ Ph.D., James R. Cook^4^ M.D., Brian J. Bolwell^5^ M.D., Ronald Sobecks^5^ M.D., Matt Kalaycio^5^ M.D., David Bosler^4^ M.D., Jaroslaw P. Maciejewski^1*^M.D., Ph.D.**

*^1^Translational Hematology and Oncology Research Department of Cleveland Clinic, USA*

*^2^Department of Biomedicine and Prevention, University of Rome Tor Vergata, Rome, Italy*

*^3^Roswell Park Comprehensive Cancer Center, Buffalo, NY*

*^4^Department of Laboratory Medicine, Cleveland Clinic, Cleveland, Ohio*

*^5^Department of Hematology and Oncology, Taussig Cancer Institute, Cleveland Clinic, Cleveland, Ohio.*

#These authors equally contributed to this work

***Correspondence:**

Jaroslaw P. Maciejewski, M.D., Ph.D., FACP

Department of Translational Hematology and Oncology Research, Taussig Cancer Institute

9620 Carnegie Ave, Building NE6-314, Cleveland, OH, USA 44106

Phone: +1 216-445-5962; E-mail: [maciejj@ccf.org](mailto:maciejj@ccf.org)

**Table of contents**

Research question, study design, objectives, and definitions………………………….....................………….....……4

Patient population…………….……………………………………………………….…............................................................6

Conventional cytogenetics…………………………...………………………...……………..…….…………………………………………7

Genomic studies……………………………...……………………………...……………...…………………………………………..…………7

Statistical analysis…..………………………………………………………………………..........................................................8

**Supplementary tables**

Supplementary Table S1. Summary of the cohorts of tMN and CH cases included in our study……....……10

Supplementary Table S2. List of 63 genes targeted in our diagnostic next-generation sequencing panel used to detect CH mutations of leukemogenic potential…….…………………………………………..…………………….11

Supplementary Table S3. Determination of ancestral, codominant, and subclonal mutations..…………....12

Supplementary Table S4. Baseline demographic and clinical characteristics of the aHSCT case-matched cases used for CH sequencing in our 1:2 case control…………………………………………………..……………………….13

Supplementary Table S5. Frequency of specific gene mutations in post-aHSCT tMN versus other tMN…14
Supplementary Table S6. Demographic and clinical characteristics of patients developing tMN after aHSCT compared to cases not registering tMN evolution………………………………..…………….........................15

Supplementary Table S7. Univariate and multivariate analysis of variables influencing the risk of tMN in patients undergoing aHSCT…………………………………………………………………..………..……………………………………..16

Supplementary Table S8. Univariate and multivariate analysis of variables influencing the risk of CH pre aHSCT………………………………………………………………………………………………...…..................................................17

Supplementary Table S9. Frequency of CH in healthy controls and solid tumor patients aged <60 or ≥60 years compared to patients undergoing aHSCT…………………..…………………………………………………………………18

Supplementary Table S10. Frequency of most common CH mutations in healthy controls and solid tumor patients compared to patients undergoing aHSCT…………….…………………………………………………………………..19

Supplementary Table S11. Sequencing results of our 1:2 case-control study.………………………....…………….20

**Supplementary Figures**

Supplementary Figure S1. Flowchart of patient inclusion and cohort comparisons performed in our study……………………………………………………………………………………………………...................................................23

Supplementary Figure S2. The frequency of cytogenetic abnormalities between post-aHSCT tMN and other tMN….….............................................................................................................................................24

Supplementary Figure S3. Frequency of somatic gene mutations in patients undergoing aHSCT…………..25

Supplementary Figure S4. Percentage of patients according to number of mutations in post-aHSCT tMN versus other tMN…………………………………………………………………………………………………………………………………..26

Supplementary Figure S5. Overall survival of post-aHSCT tMN patients according to mutational burden…………………………………………………………………………………………………………………………………………………..27

Supplementary Figure S6. Overall survival of CH-derived versus non-CH post-aHSCT tMN in patients aged ≥ 65 year…………………………………………………………………………………................................................................28

Supplementary Figure S7. Odds of developing post-aHSCT tMN according to codominant or dominant CH mutation……………………………………………………………………………………………………………………………………………….29

Supplementary Figure S8. Cumulative incidence in patients with antecedent pre-aHSCT *PPM1D* mutations, *TP53* mutations, other CH and no CH mutations..…….…………………………………….......................30

Supplementary Figure S9. Overall survival of *TP53*-related post-aHSCT tMN compared to other post-aHSCT tMN…………………………………………………………………………………………….………………………………………………31

Supplementary Figure S10. Modes of post-aHSCT tMN………………………………………………............................32

Supplementary Figure S11. Schemes and frequencies of modes of post-aHSCT tMN………………..…………..34

**References**…………………………………………………………………………..........………………………………………………………..35

**Research question, study design, objectives, and definitions**

**1. The research question: PICOT format**

*In therapy-related myeloid neoplasms (tMN) developing post-autologous stem cell transplant (aHSCT), how does antecedent clonal hematopoiesis (CH) influence the risk, molecular pathogenesis, and clinical outcomes of tMN post-aHSCT?*

P: Patients with tMN post-aHSCT and post-other cytotoxic therapies followed at The Cleveland Clinic Foundation (Cleveland, Ohio, USA) between 2010 and 2022, in addition to publicly available records of CH in healthy individuals and solid tumors^1-3^

I: Baseline demographic, clinical, cytogenetic, and molecular characteristics as risk-factors for CH pre-aHSCT and to tMN development post-aHSCT

C: Healthy controls and patients with solid tumor, in addition to tMN not deriving from antecedent aHSCT and patients who did not develop tMN-post aHSCT

O: Diagnosis of CH and myeloid neoplasm (WHO 2022) and after cytotoxic therapy^4, 5^

T: Follow-up from aHSCT or tMN diagnosis to the last visit or death

**2. Inclusion/exclusion criteria**

2.1 Inclusion criteria:

- Clinical diagnosis of tMN following aHSCT or other cytotoxic therapies
- Follow-up at our center
- No age limits
- Available clinical data
- Available genomic data or bone marrow samples for retrospective sequencing
- tMN was defined as the development of myeloid neoplasms in patients with prior cytotoxic therapy exposure as part of their treatment of primary neoplasms, as supported by the updated 2022 World Health Organization (WHO) and 2022 International Consensus Classification (ICC) criteria^5, 6^.

2.2 Exclusion criteria:

- Known personal history of myeloid neoplasms prior to cytotoxic therapy, including history of myeloid dysplasia or increased blasts
- Known personal history of paroxysmal nocturnal hemoglobinuria (PNH), monoclonal gammopathy of undetermined significance (MGUS) or monoclonal B-cell lymphocytosis (MBL).
- Lack of genetic studies or availability of stored bone marrow samples for testing

**3. Objectives**

3.1 Primary endpoint and main objective:

Cumulative incidence of tMN post-aHSCT and the CH-related clonal trajectories driving its pathogenesis

3.2 Secondary objectives:

- Clinical, cytogenetic and molecular characteristics that define post-aHSCT tMN compared to other tMN
- Clinical risk factors for therapy-related CH pre-aHSCT and for tMN post-aHSCT
- Prevalence of therapy-related CH pre-aHSCT relative to healthy controls and patients with non-hematological malignancies
- Clinical implications of antecedent CH on outcomes including tMN diagnosis and pathogenesis
- tMN latency (time from cytotoxic exposure to diagnosis) and overall survival (time from diagnosis to last follow-up or death) in post-aHSCT tMN, including CH-related and non-CH-related disease, and in other tMN
- Characterization of the genomic landscape of CH versus non-CH-related post-aHSCT tMN
- Characterization of gene-specific mutational changes through aHSCT
- Identifying specific antecedent ancestral CH mutations predictive of subsequent tMN evolution post-aHSCT

**4. Definitions:**

- tMN diagnosis was established according to standard guidelines^5^ and severity defined according to universally accepted scoring systems (when appropriate)^7^.
- tMN was defined as new diagnosis of myeloid neoplasm following cytotoxic therapy for primary malignancies according to the 2022 revision of the World Health Organization (WHO) classification of myeloid neoplasms, including therapy-related myelodysplastic syndromes (tMDS) and acute myeloid leukemias (tAML).
- Post-aHSCT tMN refers to tMN that developed after aHSCT; other tMN refers to tMN that followed chemotherapy or radiation exposures but not aHSCT.
- MDS risk was categorized according to Pfeilstöcker et al^8^ using the revised International prognostic scoring system (IPSS-R) criteria^7^. Accordingly, higher-risk MDS (HR-MDS) and lower-risk MDS (LR-MDS) were determined for IPSS-R >3.5 and ≤3.5 scores, respectively^7^. Further categorization using the Molecular International Prognostic Scoring System (IPSS-M) was performed.
- CH was defined as the presence of clonal hematopoiesis with at least 1 somatic mutation with evidence of leukemogenic potential as per the literature and variant allele frequency (VAF) of at least 2%^2, 3, 9-13^.
- Antecedent CH refers to CH detected on sequencing of bone marrow samples after induction and consolidative chemotherapy and right before aHSCT.
- Therapy-related CH describes antecedent CH that is significantly more prevalent in patients with cytotoxic exposure compared to healthy controls.
- Ancestral or dominant mutations were determined as we previously described according to clonal burden^14-16^ (**Supplementary Table S3**).
- Post-aHSCT tMN is categorized into CH-derived tMN and non-CH tMN.
- CH-derived tMN was determined by the incidence of tMN related to prior CH clone detection on sequencing pre-tMN diagnosis. It is further subcategorized into antecedent CH-derived tMN and de-novo (non-antecedent) CH-related tMN. The latter was defined as CH that was detected post-aHSCT but pre-tMN diagnosis. To note, this was only considered in patients who were sequenced as part of their clinical course (*e.g.,* ongoing cytopenia) after transplant. No active sequencing of samples after aHSCT and pre-tMN diagnosis was performed.
- Non-CH tMN was determined by new tMN with no prior CH detection pre-tMN diagnosis. CH-eliminated tMN is a subcategory of non-CH tMN in which previously detected CH-mutations were eventually eliminated and hence absent at the time of tMN diagnosis.
- Mutations were considered conserved through aHSCT if they were detected on both pre- and post-aHSCT bone marrow sampling, eliminated if detected on pre- but not post-aHSCT sampling, and gained/*de novo* if absent on pre-aHSCT but present on post-aHSCT sequencing.

**Patient population**

We retrospectively screened all patients who underwent aHSCT at The Cleveland Clinic Foundation (Cleveland, Ohio, USA) for tMN diagnosis between January 2010 and February 2022. We also accrued all consecutive tMN patients with no prior aHSCT exposure who have been followed at our institution. Data on CH in healthy controls and solid tumors was accessed from publicly available sources^1-3^. Written informed consent was obtained from each participant. The review of medical records was approved by the internal Institutional Review Board of our center in agreement with the Declaration of Helsinki . All patients had been regularly followed until February 2023 (last follow-up or death). Pertinent clinical data including age, sex, primary malignancy diagnosis, treatments including number and types of cytotoxic exposures, mobilization regimens, CD34 dose, cytogenetics, serial mutational analysis, tMN diagnosis with disease transformation, and other clinical complications were collected. CH clone was defined based on the presence of a somatic mutation of leukemogenic potential and VAF of at least 2%^9, 17^. In total, our study cohort included 1507 patients who underwent aHSCT, of whom 35 developed tMN, in addition to 263 patients with other tMN of whom 143 had available BM samples. We also performed demographic and clinical case matching of 70 patients (accodring to the variables mentioned in **Supplementary Table S4**) who did not develop tMN post-aHSCT (out of 1472) after sufficient follow up period in order to retrospectively sequence their pre-aHSCT bone marrow samples and analyze their mutational characteristics compared to post-aHSCT tMN patients in a 1:2 case-control study. Of the 35 post-aHSCT tMN patients, 31 had pre-aHSCT BM samples while 29 had post-aHSCT samples.

**Conventional Cytogenetics**

Fluorescent in situ hybridization (FISH) panel for myeloid neoplasms was performed to identify common and clinically relevant cytogenetic aberrations^18^. In addition, G-banded metaphase cytogenetic analysis using standard techniques was performed on bone marrow aspirates with a median number of metaphases analyzed of 20. Karyotypes were described according to the International System for Human Cytogenetic Nomenclature^19-21^.

**Genomic studies**

Patients were routinely tested for somatic mutations as part of their diagnostic work up for tMN using a next-generation sequencing (NGS) targeted panel for genes that are most commonly affected in myeloid neoplasms. Data on mutational characteristics were retrieved through retrospective chart review of the diagnostic work up at tMN diagnosis. This also included retrospective review of prior detected CH as part of the ongoing post-aHSCT evaluation. Patients with no NGS at tMN diagnosis were reviewed for stored bone marrow samples in our biorepository in order to perform retrospective sequencing. Our biorepository was further reviewed for other samples pre-aHSCT in order to perform progressive and sequential sequencing if available along with longitudinal evaluation of clonal dynamics. Samples were carefully selected for clinical relevance, and thosewith possible conflict of cytotoxic effects of other therapies with aHSCT were excluded. DNA was extracted from formalin fixed, paraffin embedded bone marrow clot sections. Target-enriched libraries for NGS were prepared by nested multiplex PCR-based target enrichment using anchored multiplex PCR (AMPTM, Archer® DX, Inc., Boulder, CO). Targeted coding and non-coding regions of 63 genes (**Supplementary Table S2**) were amplified and sequenced on the NextSeq NGS platform (Illumina®, San Diego, CA) with paired ends, 150x2 cycle reads. The minimum average coverage (depth) of a specimen was ≥500X, and regions with <100x coverage were not considered covered for the analysis. A customized, clinically validated bioinformatic analytical pipeline was used to map reads to the reference human genome (genome build hg19/GRCh37) and for annotation and variant detection. The variant allele fraction (VAF) filter was set to 2%. Manual review of sequencing reads for called variants was performed to aid the removal of potential artifactual calls. Variants were included in our analyses when a strong and/or potential clinical relevance was ascertained after sorting for potential artifacts and according to general population frequencies, previous literature reports, pathogenicity prediction and evaluation of serial samples.

**Statistical analyses**

Quantitative variables were expressed as medians and ranges of minimum to maximum values. Qualitative variables were reported as numbers and their relative percentages. For all relevant group comparisons, unpaired two-sided t-tests and analysis of variances (ANOVA) were performed for comparative analyses of quantitative variables. Fisher test or Chi-square were used for testing of significance for qualitative variables. Subsequent univariate analysis was reported as odds ratios (OR) along their 95% confidence intervals (CI). Cox proportional hazard models and multivariate logistic regressions were used to determine independent risks of baseline clinical and demographic characteristics on outcome events, and the results were reported as OR and Hazard ratios (HR) with their 95% CI.

Kaplan–Meier (KM) log rank testing was used to calculate and test for significance of overall survival (OS) or incidence of other events in-between groups.^22^ Overall survival (OS) was defined as the time from tMN diagnosis to event (death) or censoring (last follow-up date). We also used KM method to determine time from cytotoxic exposure to event (tMN diagnosis) or censoring (last follow-up date). Cumulative incidence of tMN was defined as the time from 1^st^ cytotoxic exposure to clinical diagnosis as evidenced by cytogenetic and molecular abnormalities with bone marrow picture of myeloid neoplasm per 2022 WHO recommendations^5^.

A p-value ≤0.05 was considered statistically significant for all types of analyses. All statistical computations were performed using R 3.6.2 (www.r-project.org) and Prism (GraphPad 9.0).

**Supplementary tables**

**Supplementary Table S1. Summary of the cohorts of tMN and CH cases included in our study.**

| Cohorts^#^ | Number of patients |
| --- | --- |
| Cleveland Clinic tMN cohorts | 298 |
| tMN post-aHSCT | 35 |
| Other tMN | 263 |
| Cleveland Clinic non-tMN aHSCT cohort | 1472 |
| Cases matched for CH sequencing | 70 |
| Publicly availably CH cohorts | 6414 |
| Healthy controls^2, 3^ | 765 |
| Solid tumors^1^ | 5649 |

**#** Only cases with available molecular studies were included.

**Supplementary Table S2. List of 63 genes targeted in our diagnostic next-generation sequencing panel used to detect CH mutations of leukemogenic potential.**

| *ABL1* | *ASXL1* | *BCOR* | *BCORL1* | *BRAF* | *CALR* |
| --- | --- | --- | --- | --- | --- |
| *CBL* | ***CDKN2A*** | ***CEBPA*** | ***CSF3R1*** | ***CUX1*** | ***DDX41*** |
| *DNMT3A* | ***EED*** | ***ETNK1*** | ***ETV6*** | ***EZH2*** | ***FBXW7*** |
| *FLT3* | ***GATA1*** | ***GATA2*** | ***GNAS*** | ***IDH1*** | ***IDH2*** |
| *IKZF1* | ***JAK2*** | ***JAK3*** | ***KDM6A*** | ***KIT*** | ***KMT2A*** |
| *KRAS* | ***LUC7L2*** | ***MPL*** | ***MYD88*** | ***NF1*** | ***NOTCH1*** |
| *NPM1* | ***NRAS*** | ***PAX5*** | ***PHF6*** | ***PIGA*** | ***PPM1D*** |
| *PRPF8* | ***PTEN*** | ***PTPN1*** | ***RAD21*** | ***RIT1*** | ***RUNX1*** |
| *SETBP1* | ***SF3B1*** | ***SH2B3*** | ***SMC1A*** | ***SMC3*** | ***SRSF2*** |
| *STAG2* | ***STAT3*** | ***STAT5B*** | ***SUZ12*** | ***TET2*** | ***TP53*** |
| *U2AF1* | ***WT1*** | ***ZRSR2*** |  |  |  |

**Supplementary Table S3. Determination of ancestral, codominant, and subclonal mutations.**

| Type | Criteria |
| --- | --- |
| Ancestral | Largest variant allele frequency on initial sampling |
| Codominant | Variant allele frequency within 5% of dominant mutation VAF (Dominant VAF%-5% ≤ Codominant VAF%) |
| Subclonal | Appears on subsequent sampling but not initial sampling  VAF% < Dominant VAF%-5% |

**Supplementary Table S4. Baseline demographic and clinical characteristics of the aHSCT case-matched cases used for CH sequencing in our 1:2 case control.**

| Variables | All | tMN | No tMN | P-value |
| --- | --- | --- | --- | --- |
| Total population | 105 | 35 | 70 |  |
| Demographics |  |  |  |  |
| Follow up, median (IQR) (months) | 35 (32-50) | 31.5 (16.5-51.7) | 35.5 (32-50) | 0.15 |
| Age at aHSCT, median (IQR) (years) | 61.6 (54.4-66.0) | 62.7 (56.4-66.4) | 61 (53.7-65.4) | 0.27 |
| <60, n (%) | 44 (41.9) | 12 (34.3) | 32 (45.7) | 0.30 |
| ≥60, n (%) | 61 (58.1) | 23 (65.7) | 38 (54.3) |  |
| Gender |  |  |  | 0.99 |
| Male, n (%) | 97 (92.4) | 32 (91.4) | 65 (92.9) |  |
| Female, n (%) | 8 (7.6) | 3 (8.6) | 5 (7.1) |  |
| Primary malignancy |  |  |  | 0.54 |
| MM, n (%) | 29 (27.6) | 9 (25.7) | 20 (28.6) |  |
| NHL, n (%) | 68 (64.8) | 23 (65.7) | 45 (64.3) |  |
| HL, n (%) | 7 (6.6) | 2 (5.7) | 5 (7.1) |  |
| Other, n (%) | 1 (1.0) | 1 (2.9) | 0 (0) |  |
| Mobilization regimen |  |  |  |  |
| G | 12 (11.4) | 4 (11.4) | 8 (11.4) | 0.99 |
| G+E | 12 (11.4) | 4 (11.4) | 8 (11.4) |  |
| G+P | 66 (62.9) | 22 (62.9) | 44 (62.9) |  |
| G+E+P | 15 (14.3) | 5 (14.3) | 10 (14.3) |  |
| Leukapheresis days |  |  |  |  |
| <4, n (%) | 79 (75.2) | 25 (71.4) | 54 (77.1) | 0.52 |
| ≥4, n (%) | 26 (24.8) | 10 (28.6) | 16 (22.9) |  |
| CD34+ dose (x10^6^/kg) |  |  |  | 0.64 |
| <3, n (%) | 27 (25.7) | 10 (28.6) | 17 (24.3) |  |
| ≥3, n (%) | 78 (74.3) | 25 (71.4) | 53 (75.7) |  |

aHSCT: autologous hematopoietic stem cell transplant, tMN: therapy-related myeloid neoplasm; IQR: interquartile range, n: number, %: percentage, MM: multiple myeloma, NHL: non-Hodgkin’s lymphoma, HL: Hodgkin’s lymphoma, G: G-CSF, E: etoposide, P: plerixafor.

**Supplementary Table S5. Frequency of specific gene mutations in post-aHSCT tMN versus other tMN.**

| Mutations | Post-aHSCT tMN, n (%) | Other tMN, n (%) |  |
| --- | --- | --- | --- |
| N | **29** | **143** | **P-value** |
| *ASXL1* | 1 (3.5) | 16 (11.2) | 0.31 |
| *BCOR* | 0 (0) | 10 (7.0) | 0.14 |
| *BCORL1* | 1 (3.5) | 3 (2.1) | 0.53 |
| *CUX1* | 1 (3.5) | 5 (3.5) | 0.99 |
| *DNMT3A* | 2 (6.9) | 16 (11.2) | 0.74 |
| *EZH2* | 1 (3.5) | 10 (7.0) | 0.69 |
| *FLT3* | 1 (3.5) | 3 (2.1) | 0.53 |
| *GATA2* | 1 (3.5) | 2 (1.4) | 0.43 |
| *IDH1* | 0 (0) | 1 (0.7) | 0.99 |
| *IDH2* | 0 (0) | 3 (2.1) | 0.99 |
| *JAK2* | 0 (0) | 2 (1.4) | 0.99 |
| *KRAS* | 2 (6.9) | 4 (2.8) | 0.27 |
| *NPM1* | 0 (0) | 9 (6.3) | 0.36 |
| *NRAS* | 1 (3.5) | 6 (4.2) | 0.99 |
| *PPM1D* | 6 (20.7) | 7 (4.9) | 0.003 |
| *RAD21* | 0 (0) | 5 (3.5) | 0.59 |
| *RUNX1* | 3 (10.3) | 11 (7.8) | 0.64 |
| *SETBP1* | 1 (3.5) | 6 (4.2) | 0.99 |
| *SF3B1* | 2 (6.9) | 5 (3.5) | 0.34 |
| *SRSF2* | 0 (0) | 15 (10.5) | 0.08 |
| *STAG2* | 1 (3.5) | 11 (7.8) | 0.69 |
| *TET2* | 5 (17.2) | 25 (17.5) | 0.99 |
| *TP53* | 12 (41.4) | 18 (12.6) | <0.001 |
| *U2AF1* | 1 (3.5) | 11 (7.8) | 0.69 |
| *WT1* | 1 (3.5) | 3 (2.1) | 0.53 |
| *ZRSR2* | 1 (3.5) | 3 (2.1) | 0.53 |

aHSCT: autologous hematopoietic stem cell transplant, tMN: therapy-related myeloid neoplasm, %: percentage, N: number.

**Supplementary Table S6.** **Demographic and clinical characteristics of patients developing tMN after aHSCT compared to cases not registering tMN evolution.**

| Variables | All aHSCT | tMN | No tMN |
| --- | --- | --- | --- |
|  | N=1507 | N=35 | N=1472 |
| Demographics |  |  |  |
| Age in years at aHSCT, median (IQR) | 59.4 (50.7-65.6) | 62.7 (56.4-66.4) | 59.2 (50.4-65.3) |
| <60, n (%) | 1090 (72.3) | 12 (34.3) | 792 (53.8) |
| ≥60, n (%) | 417 (27.7) | 23 (65.7) | 680 (46.2) |
| Gender, n (%) |  |  |  |
| Male | 871 (57.8) | 32 (91.4) | 839 (56.7) |
| Female | 636 (41.2) | 3 (8.6) | 633 (43.3) |
| Primary malignancy, n (%) |  |  |  |
| MM | 790 (53.4) | 9 (25.7) | 781 (53.1) |
| NHL | 519 (46.6) | 23 (65.7) | 496 (33.7) |
| HL | 136 (9.0) | 2 (5.7) | 134 (9.1) |
| Brain | 32 (2.1) | 1 (2.9) | 31 (2.1) |
| Testicular germline | 23 (1.5) | 0 (0) | 23 (1.6) |
| Bone | 7 (0.4) | 0 (0) | 7 (0.4) |
| Chemotherapy lines, n (%) |  |  |  |
| 1 | 766 (50.8) | 8 (22.9) | 758 (51.5) |
| 2 | 624 (41.4) | 15 (42.9) | 609 (41.4) |
| ≥3 | 117 (7.8) | 12 (34.2) | 105 (7.1) |
| Radiation therapy, n (%) | 103 (6.8) | 13 (37.1) | 90 (6.1) |
| Mobilization regimen, n (%) |  |  |  |
| G | 240 (15.9) | 4 (11.4) | 236 (16.0) |
| G+E | 117 (7.8) | 4 (11.4) | 113 (7.7) |
| G+P | 1078 (71.5) | 22 (62.9) | 1056 (71.7) |
| G+E+P | 42 (2.8) | 5 (14.3) | 37 (2.5) |
| Other | 30 (2.0) | 0 (0) | 30 (2.1) |
| Leukapheresis length in days, n (%) |  |  |  |
| <4 | 1198 (79.5) | 25 (71.4) | 1173 (79.7) |
| ≥4 | 309 (20.5) | 10 (28.6) | 299 (20.3) |
| Collected CD34+ dose (x10^6^/kg), n (%) |  |  |  |
| <3 | 216 (14.3) | 10 (28.6) | 206 (14.0) |
| ≥3 | 1291 (85.7) | 25 (71.4) | 1266 (86.0) |

aHSCT: autologous hematopoietic stem cell transplant, tMN: therapy-related myeloid neoplasm; IQR: interquartile range, n: number, %: percentage, MM: multiple myeloma, NHL: non-Hodgkin’s lymphoma, HL: Hodgkin’s lymphoma, G: G-CSF, E: etoposide, P: plerixafor.

**Supplementary Table S7. Univariate and multivariate cox proportional hazar ratios of variables influencing the risk of tMN in patients undergoing aHSCT.**

| Variables | Univariable | | Multivariable | |
| --- | --- | --- | --- | --- |
|  | **HR (95% CI)** | **P-value** | **HR (95% CI)** | **P-value** |
| Age ≥ 60 years | 2.3 (1.1-4.6) | 0.02 | 2.5 (1.2-5.3) | 0.01 |
| Male sex | 7.5 (2.3-24.6) | <0.001 | 6.3 (1.9-20.9) | 0.003 |
| Primary malignancy |  |  |  |  |
| NHL | 3.7 (0.9-15.7) | 0.07 | 2.2 (0.5-9.9) | 0.32 |
| MM | 0.9 (0.2-4.2) | 0.90 | 0.6 (0.1-3.1) | 0.52 |
| Mobilization regimen |  |  |  |  |
| G+E | 1.7 (0.4-7.0) | 0.43 | 1.1 (0.3-4.7) | 0.89 |
| G+P | 1.1 (0.4-3.3) | 0.81 | 1.0 (0.3-2.8) | 0.94 |
| G+E+P | 6.2 (1.7-22.9) | 0.007 | 2.8 (0.7-11.0) | 0.15 |
| Chemotherapy ≥3 lines | 5.6 (2.8-11.4) | <0.001 | 4.7 (2.2-10.0) | <0.001 |
| Radiation | 6.0 (3.0-11.9) | <0.001 | 5.2 (2.5-10.9) | <0.001 |
| Leukapheresis days ≥4 | 1.6 (0.8-3.2) | 0.29 | 1.2 (0.4-3.0) | 0.09 |
| CD34+ dose <3.0 (x10^6^/kg) | 2.3 (1.1-5.0) | 0.03 | 2.5 (1.1-5.5) | 0.02 |

HR: hazard ratio, CI: confidence interval, NHL: non-Hodgkin’s lymphoma, HL: Hodgkin’s lymphoma, MM: multiple myeloma, G: G-CSF, E: etoposide, P: plerixafor.

**Supplementary Table S8. Univariate and multivariate analysis of variables influencing the risk of CH pre-aHSCT.**

| Variables | Univariable | | Multivariable | |
| --- | --- | --- | --- | --- |
|  | **OR (95% CI)** | **P-value** | **OR (95% CI)** | **P-value** |
| Age ≥ 60 years | 5.4 (1.7-15.9) | 0.003 | 10.4 (2.4-64.2) | 0.005 |
| Male sex | 0.4 (0.1-2.0) | 0.37 | 0.8 (0.1-5.9) | 0.86 |
| Primary malignancy |  |  |  |  |
| NHL | 0.6 (0.2-1.6) | 0.26 | 0.5 (0.1-11.2) | 0.57 |
| MM | 2.4 (0.9-6.4) | 0.11 | 1.0 (0.1-25.3) | 0.99 |
| Chemotherapy ≥ 3 lines | 3.5 (1.3-10.1) | 0.03 | 7.5 (2.1-33.5) | 0.004 |
| Radiation | 1.6 (0.6-4.5) | 0.56 | 2.5 (0.6-10.3) | 0.2 |

OR: odds ratio, CI: confidence interval, NHL: non-Hodgkin’s lymphoma, HL: Hodgkin’s lymphoma, MM: multiple myeloma.

**Supplementary Table S9. Frequency of CH in healthy controls and solid tumor patients aged <60 or ≥60 years compared to patients undergoing aHSCT.**

| Cohorts | Age <60 years | | Age ≥60 years | |
| --- | --- | --- | --- | --- |
|  | **Frequency (%)** | **P-value** | **Frequency (%)** | **P-value** |
| Healthy controls^2, 3^ | 7.9 | 0.3 | 17.9 | <0.001 |
| Pre-aHSCT | 12.5 | NA | 43.8 | NA |
| Solid tumors^1*^ | 13.4 | 0.82 | 33.4 | 0.11 |
| Biliary | 8.7 | 0.62 | 38.5 | 0.48 |
| Bladder | 14.5 | 0.75 | 41.7 | 0.71 |
| Breast | 13.8 | 0.78 | 33.8 | 0.15 |
| Colorectal | 13.2 | 0.86 | 32 | 0.10 |
| Esophagogastric | 16.5 | 0.55 | 28.8 | 0.06 |
| Glioma | 10.2 | 0.74 | 35 | 0.28 |
| Hepatocellular | 6.1 | 0.39 | 39.3 | 0.65 |
| Lung | 13.1 | 0.88 | 33.9 | 0.14 |
| Melanoma | 10.7 | 0.83 | 30.9 | 0.08 |
| Mesothelioma | 0 | 0.21 | 14.1 | <0.001 |
| Ovarian | 15.9 | 0.62 | 27.9 | 0.06 |
| Pancreatic | 14.6 | 0.73 | 40 | 0.58 |
| Prostate | 11.8 | 0.96 | 34.4 | 0.18 |
| Renal cell | 12.2 | 0.99 | 25.6 | 0.02 |
| Soft tissue sarcoma | 13.9 | 0.79 | 29.2 | 0.07 |
| Thyroid | 25.9 | 0.12 | 52.5 | 0.40 |
| Uterine | 20 | 0.33 | 28.2 | 0.04 |

*Sequenced after tumor treatment
CH: clonal hematopiesis of indeterminate potential, aHSCT: Autologous hematopoietic stem cell transplant, %: percentage.

**Supplementary Table S10. Frequency of most common CH mutations in healthy controls and solid tumor patients compared to patients undergoing aHSCT.**

| Mutations |  | Cohorts | | | |
| --- | --- | --- | --- | --- | --- |
|  | **Pre-aHSCT** | **Healthy controls^2, 3^** | | **Solid tumors^1^** | |
|  | **Frequency (%)** | **Frequency (%)** | **P-value** | **Frequency (%)** | **P-value** |
| *ASXL1* | 1.3 | 0.8 | 0.5 | 1.8 | 0.73 |
| *DNMT3A* | 11.3 | 7.5 | 0.27 | 10.5 | 0.83 |
| *JAK2* | 1.3 | 0.4 | 0.33 | NA | NA |
| *PPM1D* | 8.8 | 0 | <0.001 | 3.4 | 0.02 |
| *TET2* | 5.0 | 3.0 | 0.31 | 3.6 | 0.54 |
| *TP53* | 5.0 | 0.1 | <0.001 | 1.1 | 0.002 |
| Other | 35 | 2.0 | <0.001 | 19.9 | 0.001 |

CH: clonal hematopoiesis of indeterminate potential, aHSCT: Autologous hematopoietic stem cell transplant, %: percentage.

**Supplementary Table S11. Sequencing results of our 1:2 case-control study.**

| UPN | Mutations Pre-aHSCT | | | | Mutations at tMN diagnosis | | | |
| --- | --- | --- | --- | --- | --- | --- | --- | --- |
|  | **Gene** | **AA change** | **cDNA change** | **VAF** | **Gene** | **AA change** | **cDNA change** | **VAF** |
| tMN post-aHSCT patients | | | | | | | | |
| #1 | *None* |  |  |  | *TET2*  *TET2*  *NRAS*  *NRAS*  *NRAS*  *NRAS* | p.P761Lfs*52 p.Q764Pfs*5  p.Q61H  p.Q61H  p.Q61R  p.Q61K | c.2280delT  c.2290dupC  c.183A>T  c.183A>C  c.182Z>G  c.181C>A | 41.9%  44%  5.2%  4.3% 10.4%  6% |
| #2 | *PPM1D* | p.R572* | c.1745C>T | 9.8% | *unavailable* |  |  |  |
| #3 | *TP53*  *TP53*  *KMT2A* | p.R213G  p.N263263Ifs*82  p.N3906S | c.637C>G  c.788del  c.11717A>G | 4.9%  4.9%  2.3% | *TP53*  *TP53* | p.R213G p.N263 Ifs*82 | c.637C>G  c.788del | 33.1%  27.1% |
| #4 | *None* |  |  |  | *TP53* | p.? | c.559+1G>A | 19.9% |
| #5 | *None* |  |  |  | *TP53*  *KRAS* | p.C135G  p.Q61H | c.403T>G  c.183A>C | 21%  2% |
| #6 | *None* |  |  |  | *PPM1D*  *RUNX1* | p.R552*  p.F163C | c.1654C>T  c.488T>G | 42.2%  40.8% |
| #7 | *None* |  |  |  | *EZH2*  *PTPN11*  *RUNX1*  *TET2*  *TET2* | p.R288L  p.G503R  p.N146Afs*16  p.F1368Sfs*34  p.L199Ffs*8 | c.863G>T  c.1507G>A  c.429_435dup  c.4091_4094dup  c.597del | 65.1%  37.9%  30.8%  38.2%  46.1% |
| #8 | *None* |  |  |  | *TP53* | p.L265R | c.794T>G | 11.8% |
| #9 | *None* |  |  |  | *none* |  |  |  |
| #10 | *PPM1D* | p.L450* | c.1349del | 11.4% | *PPM1D* | p.L450* | c.1349del | 9.5% |
| #11 | *None* |  |  |  | *TET2*  *TET2*  *GATA2* | p.S1324Kfs*36  p.S1848*  p.A318D | c.3970_3979del10  c.5543C>G  c.953C>A | 47.1% 50.7%  5.5% |
| #12 | *TP53*  *TP53* | p.P219S  p.Y220C | c.655C>T  c.659A>G | 8%  3.7% | *unavailable* |  |  |  |
| #13 | *ZRSR2*  *ZRSR2*  *SF3B1*  *TET2* | p.Q80*  p.T175C  p.A625C  p.Q1624* | c.238C>T  c.524A>G  c.1873C>T  c.4870C>T | 6.4%  3.6%  2.7%  2.3% | *ZRSR2*  *ZRSR2*  *SETBP1*  *TET2*  *PPM1D*  *RUNX1*  *RUNX1*  *FLT3*  *ASXL1* | p.Q80*  p.T175C  p.G870S  p.Q1624*  p.A523Sfs*5  p.G165C  p.L58Wfs*5  p.D835Y  p.G646Wfs*12 | c.238C>T  c.524A>G  c.2608G>A  c.4870C>T  c.1566dupA  c.493G>T  c.166_193del  c.2503G>T  c.1934dupG | 26.3%65.8%  34.3%  35.8  15.6%  11.4%  6.9%  19.7%  18.8% |
| #14 | *None* |  |  |  | *PPM1D* | p.R552* | c.1654C>T | 6.1% |
| #15 | *None* |  |  |  | *none* |  |  |  |
| #16 | *BCORL1*  *BCORL1* | p.V1564L  p.S1561I | c.4690G>C  c.4682G>T | 9.7%  9.6% | *BCORL1* | p.S1561I | c.4682G>T | 8.3% |
| #17 | *None* |  |  |  | *none* |  |  |  |
| #18 | *KDM6A* | p.? |  | 38.3% | *TP53* | p.R280T | c.839G>C | 5% |
| #19 | *TP53*  *PPM1D*  *PPM1D* | p.V216M  p.E472*  p.E447* | c. .646G>A  c.1414G>T  c.1339G>T | 6.7%  3.3% 3.1% | *TP53*  *PPM1D*  *PPM1D* | p.V216M  p.E472*  p.E447* | c. .646G>A  c.1414G>T  c.1339G>T | 92.5%  3.6%  2.3% |
| #20 | *None* |  |  |  | *PPM1D* | p.M521* | c.1561delA | 40% |
| #21 | *None* |  |  |  | *SF3B1* | p.K666N | c.1998G>C | 7.2% |
| #22 | *None* |  |  |  | *TP53* | p.P151S | c.451C>T | 77.6% |
| #23 | *None* |  |  |  | *TP53* | p.R175H | c.524G>A | 23% |
| #24 | *PPM1D* | p.C478* | c.1434C>A | 3.5% | *U2AF1* | p.S34F | c.101C>T | 27.2% |
| #25 | *None* |  |  |  | *IKZF1*  *WT1* | p.R83*  p.R380Efs*5 | c.247C>T  c.1138delinsGA | 15.5%  6.5% |
| #26 | *None* |  |  |  | *CUX1*  *DNMT3A*  *TP53* | p.S868_G870dup  p.?  p.R248W | c.2598_2606dup  c.742C>T | 19.8%  4.9%  4.6% |
| #27 | *DNMT3A* | p.R882H | c.2645G>A | 10.4% | *DNTM3A* | p.R882H | c.2645G>A | 27.2% |
| #28 | *None* |  |  |  | *TP53* | p.H214Qfs*2 | c.641dupA | 77.6% |
| #29 | *None* |  |  |  | *TP53* | p.R248G | c.742C>G | 38.4% |
| #30 | *SMC1A* | p.R711W | c.2131C>T | 43.5% | *SMC1A* | p.R711W | c.2131C>T | 74.8% |
| #31 | *None* |  |  |  | *KRAS*  *TET2* | p.Q61H  p.K793* | c.183A>C  c.2377A>T | 44%  41% |
| Control group (patients without tMN post-aHSCT) | | | | | | | | |
| #32 | *None* |  |  |  |  |  |  |  |
| #33 | *None* |  |  |  |  |  |  |  |
| #34 | *None* |  |  |  |  |  |  |  |
| #35 | *None* |  |  |  |  |  |  |  |
| #36 | *None* |  |  |  |  |  |  |  |
| #37 | *None* |  |  |  |  |  |  |  |
| #38 | *None* |  |  |  |  |  |  |  |
| #39 | *DNMT3A* | p.W860R | c.2578T>C | 4.6% |  |  |  |  |
| #40 | *DNMT3A*  *GATA2*  *PPM1D* | p.R729W  p.A411V  p.E472* | c.2185C>T  c.1232C>T  c.1414G>T | 7.6%  49.1%  6.2% |  |  |  |  |
| #41 | *None* |  |  |  |  |  |  |  |
| #42 | *None* |  |  |  |  |  |  |  |
| #43 | *DNMT3A*  *DNMT3A*  *JAK2*  *PPM1D*  *TET2*  *TET2*  *TET2*  *TP53*  *TP53* | p.P777H  p.F354Vfs*55  p.V617F  p.C478*  p.I1873T  p.N1277Ifs*86  p.?  p.Y220C  p.G266Q | c.2330C>A  c.1054_1058dup  c.1849G>T  c.1434C>A  c.5618T>C  c.3829del  c.659A>G  c.797G>A | 46.9% 4.8%  6.2%  31.1%  51.9%  42.2%  3%  6.6  2.5% |  |  |  |  |
| #44 | *None* |  |  |  |  |  |  |  |
| #45 | *None* |  |  |  |  |  |  |  |
| #46 | *BCOR*  *BCOR*  *KDM6A*  *NF1* | p.?  p.P601S  p.P938S  p.? | c.4072-1G>A  c.1801C>T  c.2812C>T  c.7394+1G>A | 2.7%  2.3%  2.6%  12.4% |  |  |  |  |
| #47 | *None* |  |  |  |  |  |  |  |
| #48 | *None* |  |  |  |  |  |  |  |
| #49 | *None* |  |  |  |  |  |  |  |
| #50 | *None* |  |  |  |  |  |  |  |
| #51 | *None* |  |  |  |  |  |  |  |
| #52 | *None* |  |  |  |  |  |  |  |
| #53 | *None* |  |  |  |  |  |  |  |
| #54 | *PPM1D*  *PPM1D* | p.L484*  p.T537Hfs*2 | c.1451del  c.1608del | 16.4%  5.7% |  |  |  |  |
| #55 | *None* |  |  |  |  |  |  |  |
| #56 | *DNMT3A*  *TET2* | p.Q842R  p.Q1030* | c.2525A>G  c.3088C>T | 3.9%  5.2% |  |  |  |  |
| #57 | *None* |  |  |  |  |  |  |  |
| #58 | *None* |  |  |  |  |  |  |  |
| #59 | *None* |  |  |  |  |  |  |  |
| #60 | *None* |  |  |  |  |  |  |  |
| #61 | *DNMT3A*  *RUNX1* | p.V657M  p.S218N | c.1969G>A  c.653G>A | 5.2%  2.9% |  |  |  |  |
| #62 | *DNMT3A*  *DNMT3A* | p.T645A  p.? | c.1933A>G  c.1123-2A>C | 14.7%10.9% |  |  |  |  |
| #63 | *None* |  |  |  |  |  |  |  |
| #64 | *TET2* | p.S231Vfs*23 | c.689dup | 4.9% |  |  |  |  |
| #65 | *None* |  |  |  |  |  |  |  |
| #66 | *None* |  |  |  |  |  |  |  |
| #67 | *None* |  |  |  |  |  |  |  |
| #68 | *None* |  |  |  |  |  |  |  |
| #69 | *SF3B1* | p.L700E | c.2098A>G | 3.3% |  |  |  |  |
| #70 | *ASXL1* | p.G967del | c.2898_2900del | 53.6% |  |  |  |  |
| #71 | *None* |  |  |  |  |  |  |  |
| #72 | *None* |  |  |  |  |  |  |  |
| #73 | *None* |  |  |  |  |  |  |  |
| #74 | *None* |  |  |  |  |  |  |  |
| #75 | *None* |  |  |  |  |  |  |  |
| #76 | *None* |  |  |  |  |  |  |  |
| #77 | *NRAS* | p.G12S | c.34G>A | 3.2% |  |  |  |  |
| #78 | *None* |  |  |  |  |  |  |  |
| #79 | *DNMT3A* | p.P904L | c.2711C>T | 5.2% |  |  |  |  |
| #80 | *DNMT3A* | p.L859S | c.2576T>C | 2.2% |  |  |  |  |

UPN: unidentified patient number; aHSCT: autologous hematopoietic stem cell transplant; t-MN: therapy-related myeloid neoplasm; AA: amino acid; VAF: variant allele frequency

**Supplementary Figures**

**Supplementary Figure S1. Flowchart of patient inclusion and cohort comparisons performed in our study.**


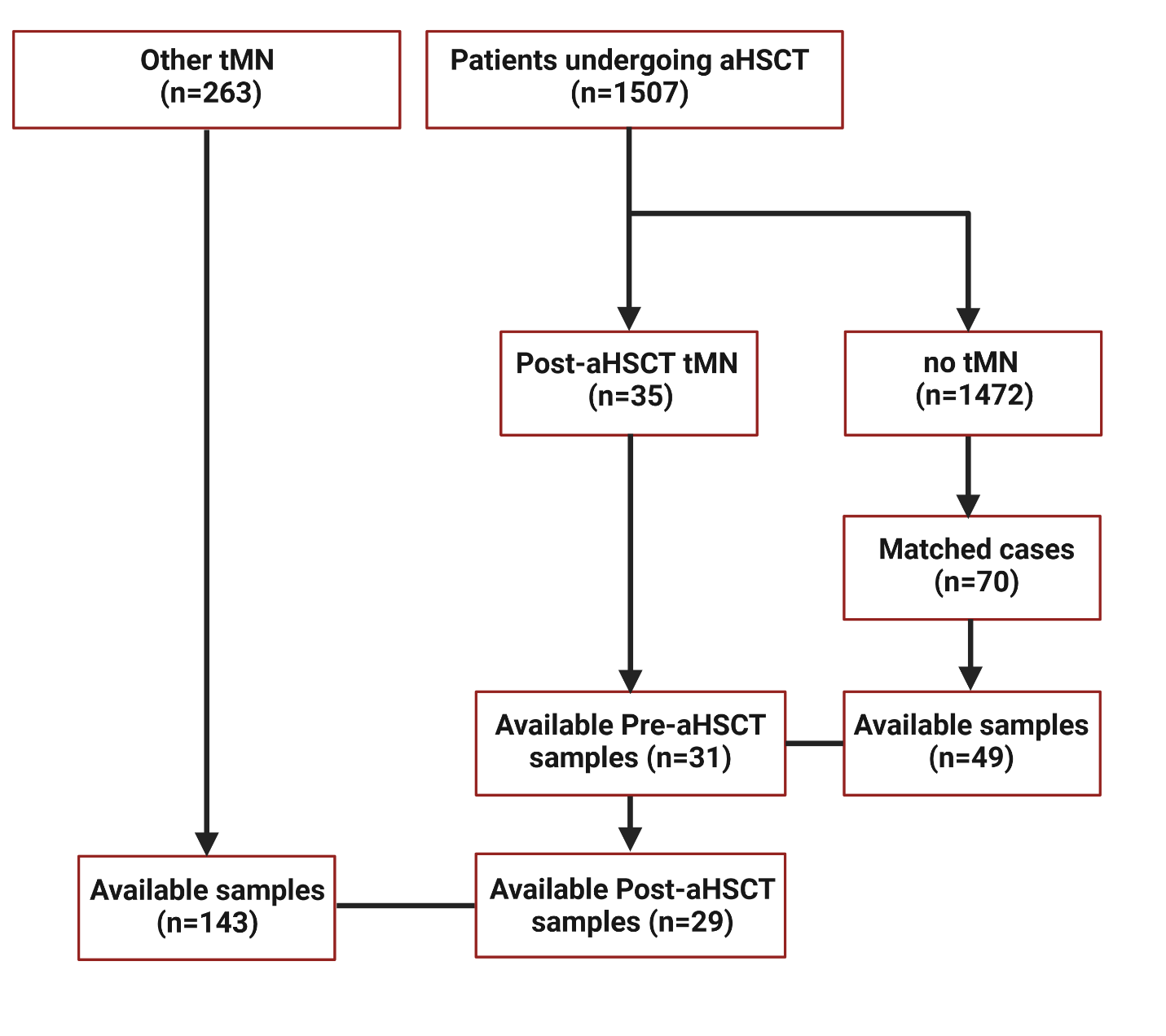


tMN: therapy-related myeloid neoplasm, aHSCT: autologous hematopoietic stem cell transplant, N: number.

**Supplementary Figure S2. The frequency of cytogenetic abnormalities between post-aHSCT tMN and other tMN.**


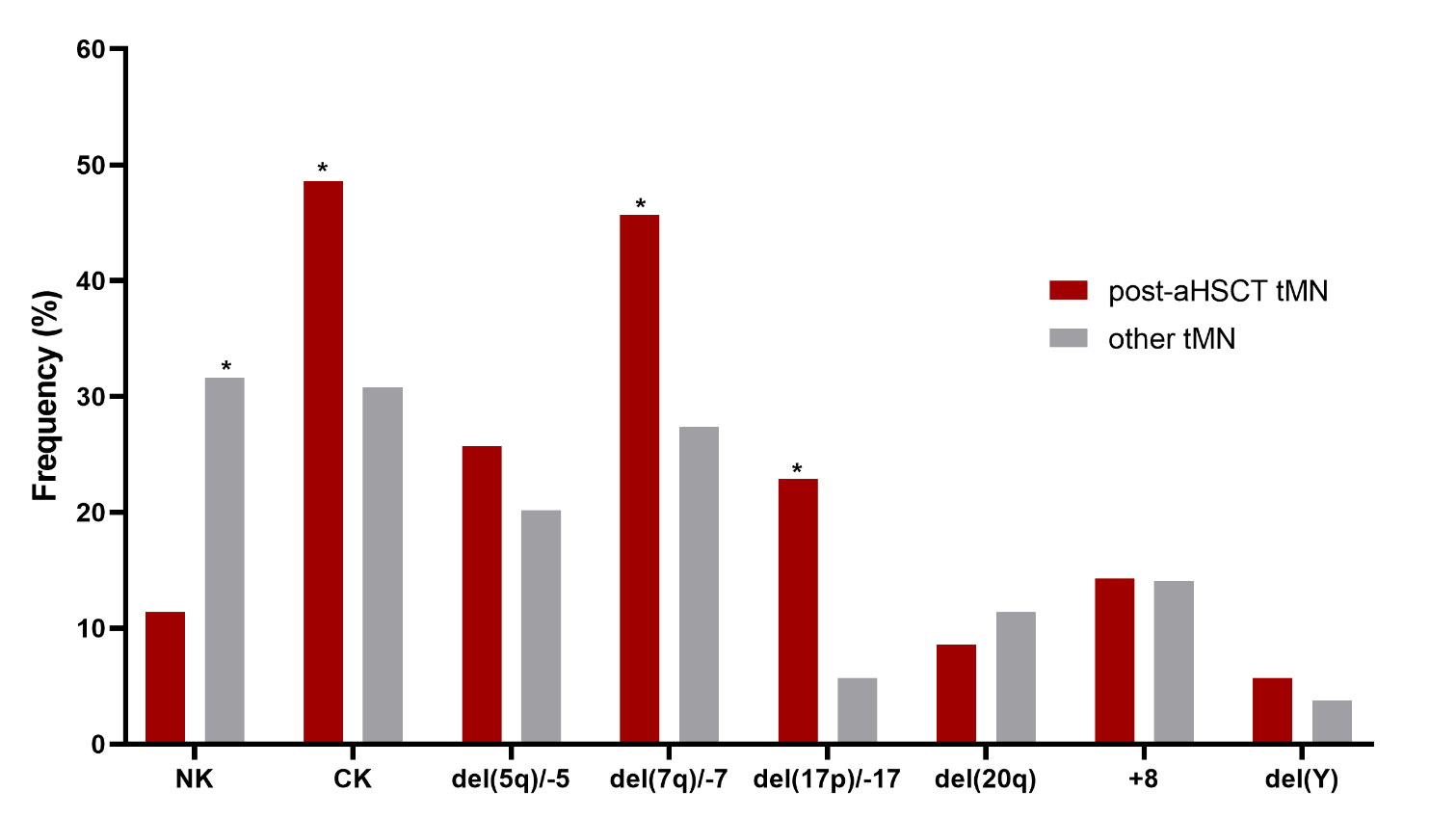


aHSCT: autologous hematopoietic stem cell transplant, tMN: therapy-related myeloid neoplasm, %:percentage, NK: normal karyotype, CK: complex karyotype, del: deletion, (-): monosomy, (+): trisomy.

**Supplementary Figure S3. Frequency of somatic gene mutations in patients undergoing aHSCT.**

**
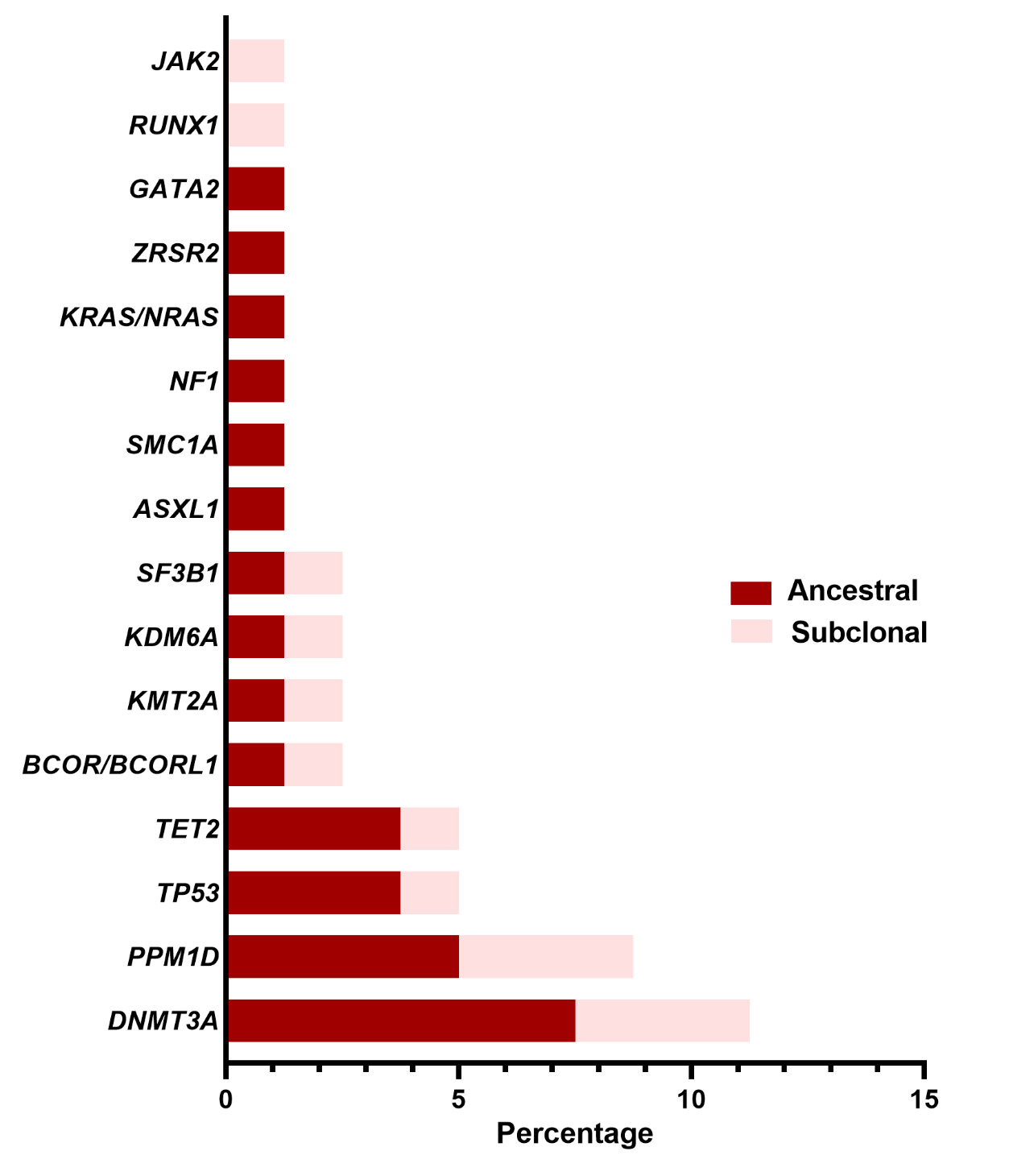
**

**Supplementary Figure S4. Percentage of patients according to number of mutations in post-aHSCT tMN versus other tMN.**

**
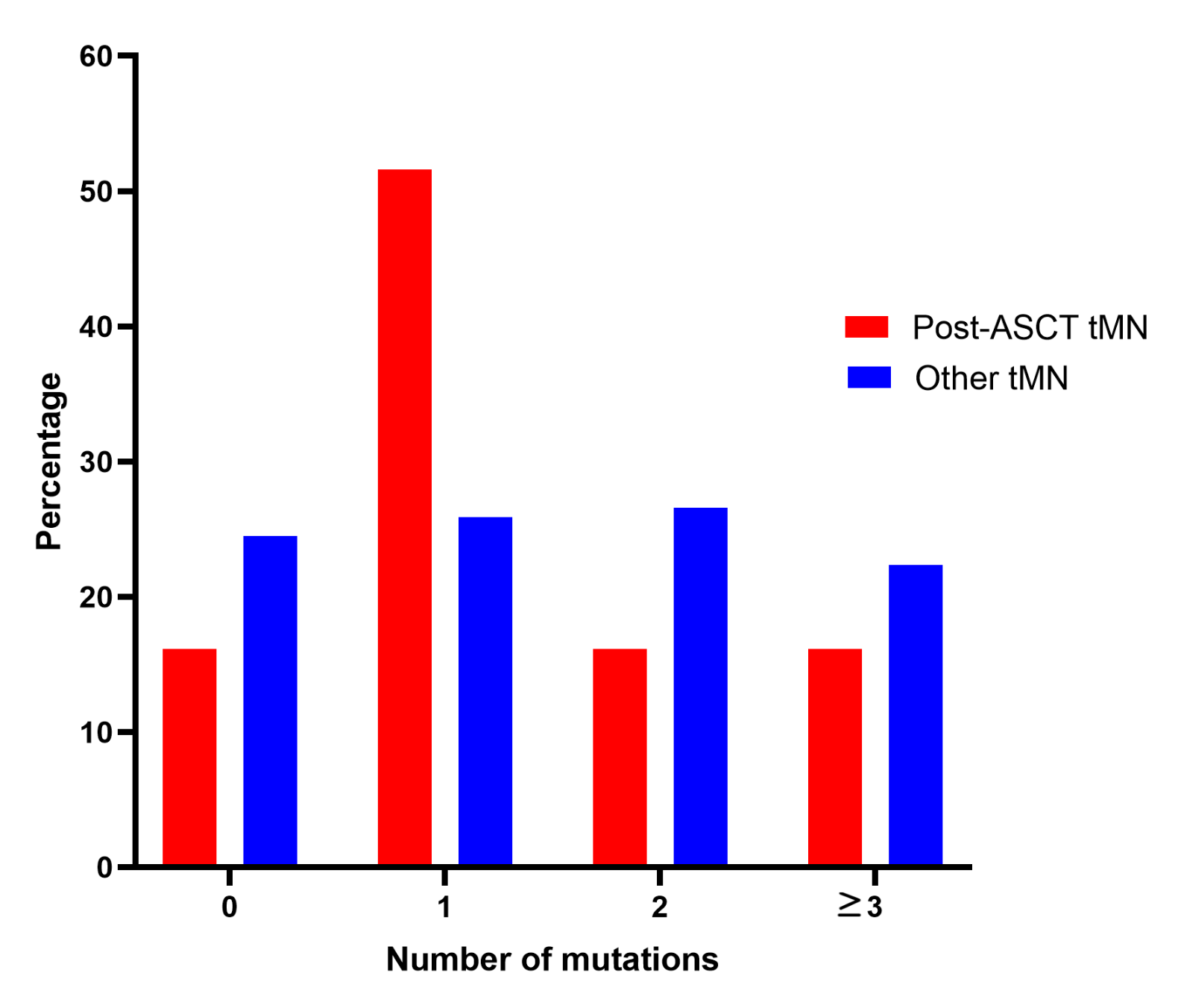
**

**post-aHSCT tMN**

**Other tMN**

aHSCT : autologous hematopoietic stem cell transplant, tMN : therapy-related myeloid neoplasm

**Supplementary Figure S5. Overall survival of post-aHSCT tMN patients according to mutational burden.**

**
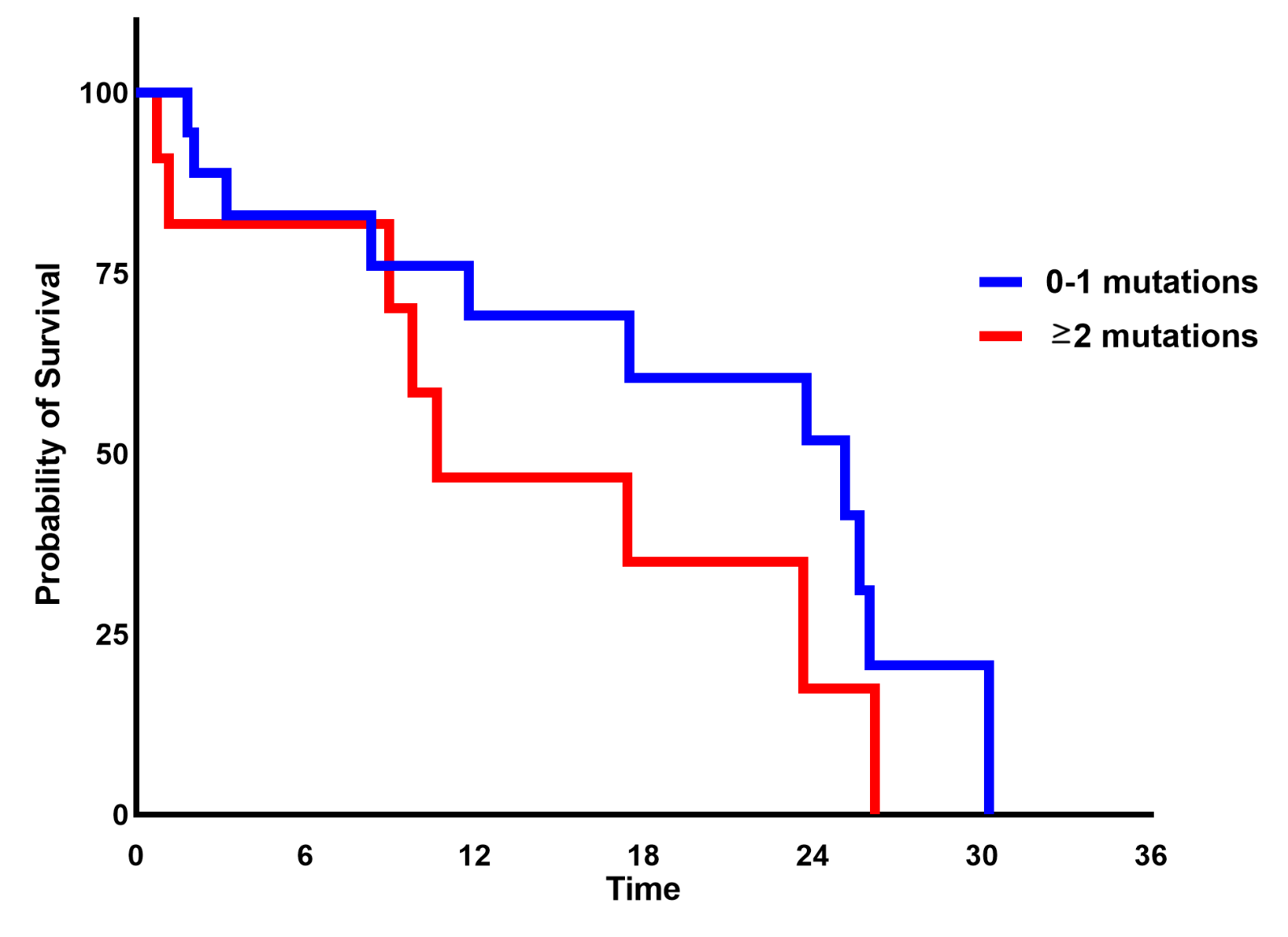
**

**(months)**

Kaplan-Meier curve shows overall survival (OS) defined as the time from tMN diagnosis to death for any cause. Censoring was applied to cases alive at the last follow-up. Median OS for patients with 0-1 mutations was 25.1 versus 10.7 months in patients with ≥ 2 mutations (P=0.16).

**Supplementary Figure S6. Overall survival of CH-derived versus non-CH post-aHSCT tMN in patients aged ≥ 65 years.**

**
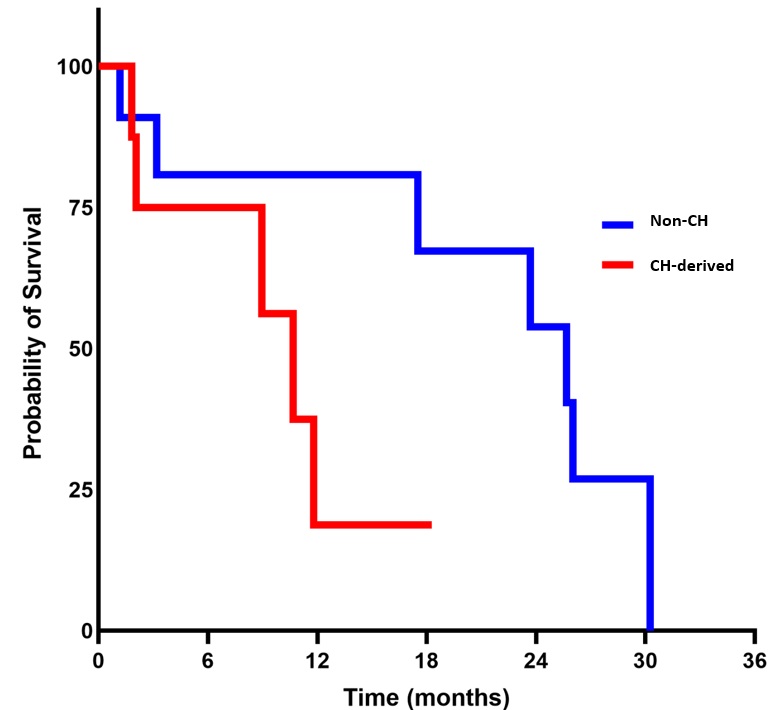
**

**Non-CH

CH-derived**

Kaplan-Meier curve shows overall survival (OS) defined as the time from tMN diagnosis to death for any cause in patients diagnosed at age ≥ 65 years. Censoring was applied to cases alive at the last follow-up. Median OS for patients with CH-derived disease was 10.7 versus 25.7 months in patients with Non-CH disease (P=0.07).

**Supplementary Figure S7. Odds of developing post-aHSCT tMN according to codominant or dominant CH mutation.**

**
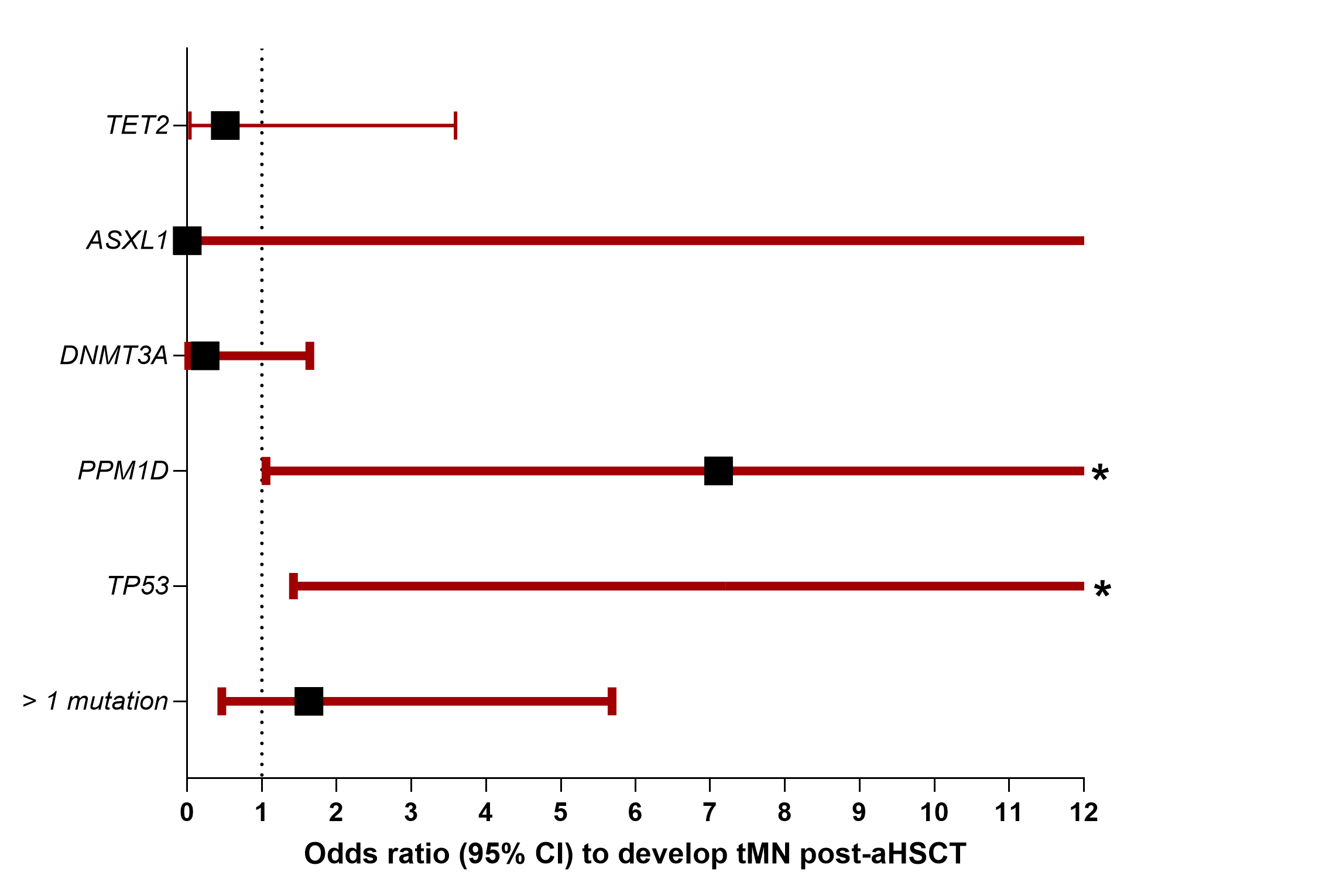
**

**Supplementary Figure S8. Cumulative incidence in patients with antecedent pre-aHSCT *PPM1D* mutations, *TP53* mutations, other CH and no CH mutations.**

**
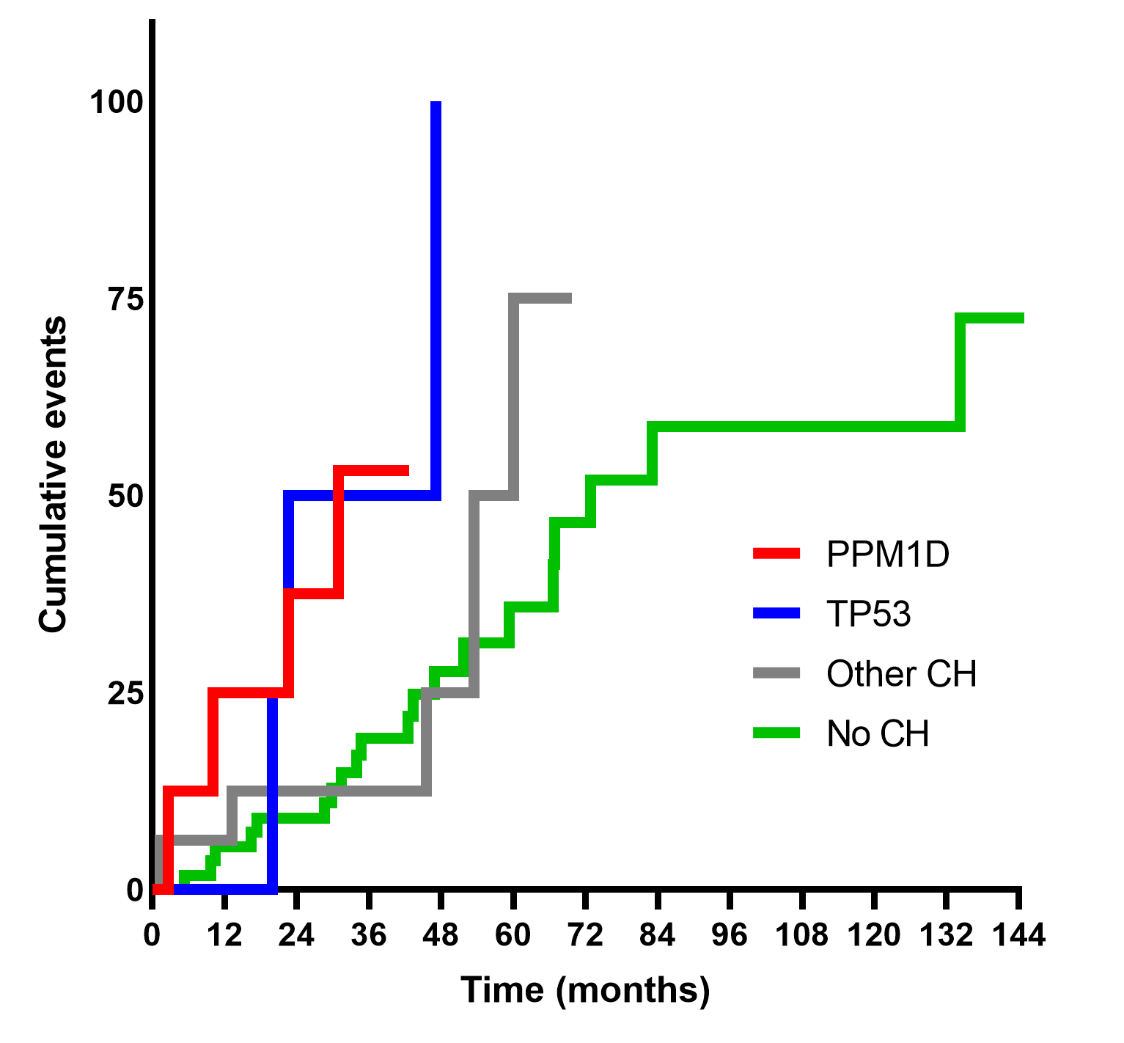
**

Reversed Kaplan-Meier curves showing time to tMN defined as the time from aHSCT to tMN diagnosis (event). Censoring was applied to cases alive at the last follow-up. Median time to tMN for patients with pre-aHSCT mutations affecting *PPM1*D or *TP53* was significantly shorter compared to other CH mutations or no CH (median 30.9 and 34.9 vs 56.8 and 72.8 months, respectively, P=0.01).

**Supplementary Figure S9. Overall survival of TP53-related post-aHSCT tMN compared to other post-aHSCT tMN.**

**
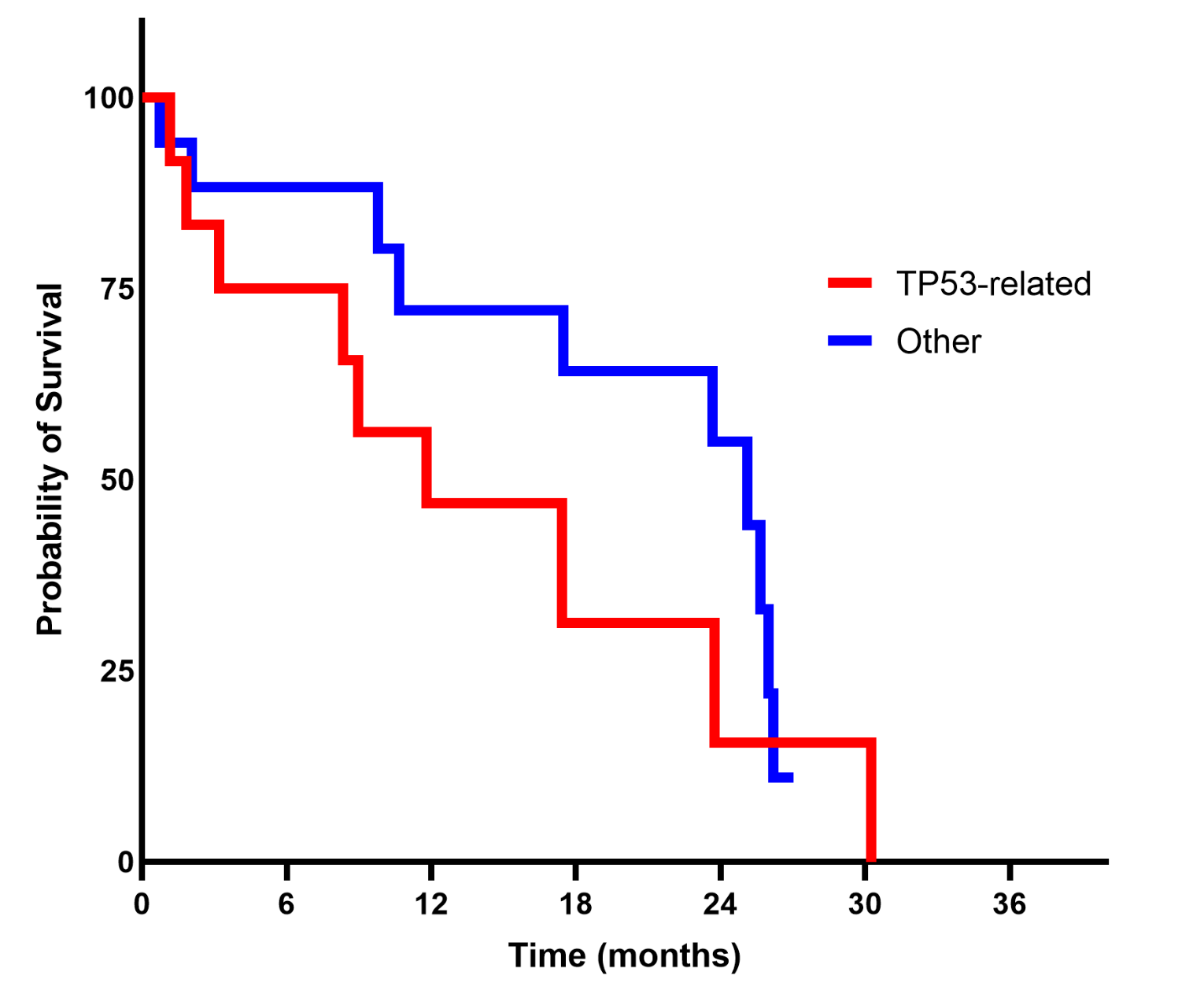
**

Kaplan-Meier curves showing overall survival (OS) defined as the time from tMN diagnosis to death (event). Censoring was applied to cases alive at the last follow-up. Median OS for patients with *TP53*-related post-aHSCT tMN was shorter than other post-aHSCT tMN cases (11.8 vs 25.1 months, P=0.28).

**Supplementary Figure S10. Modes of post-aHSCT tMN.**

**
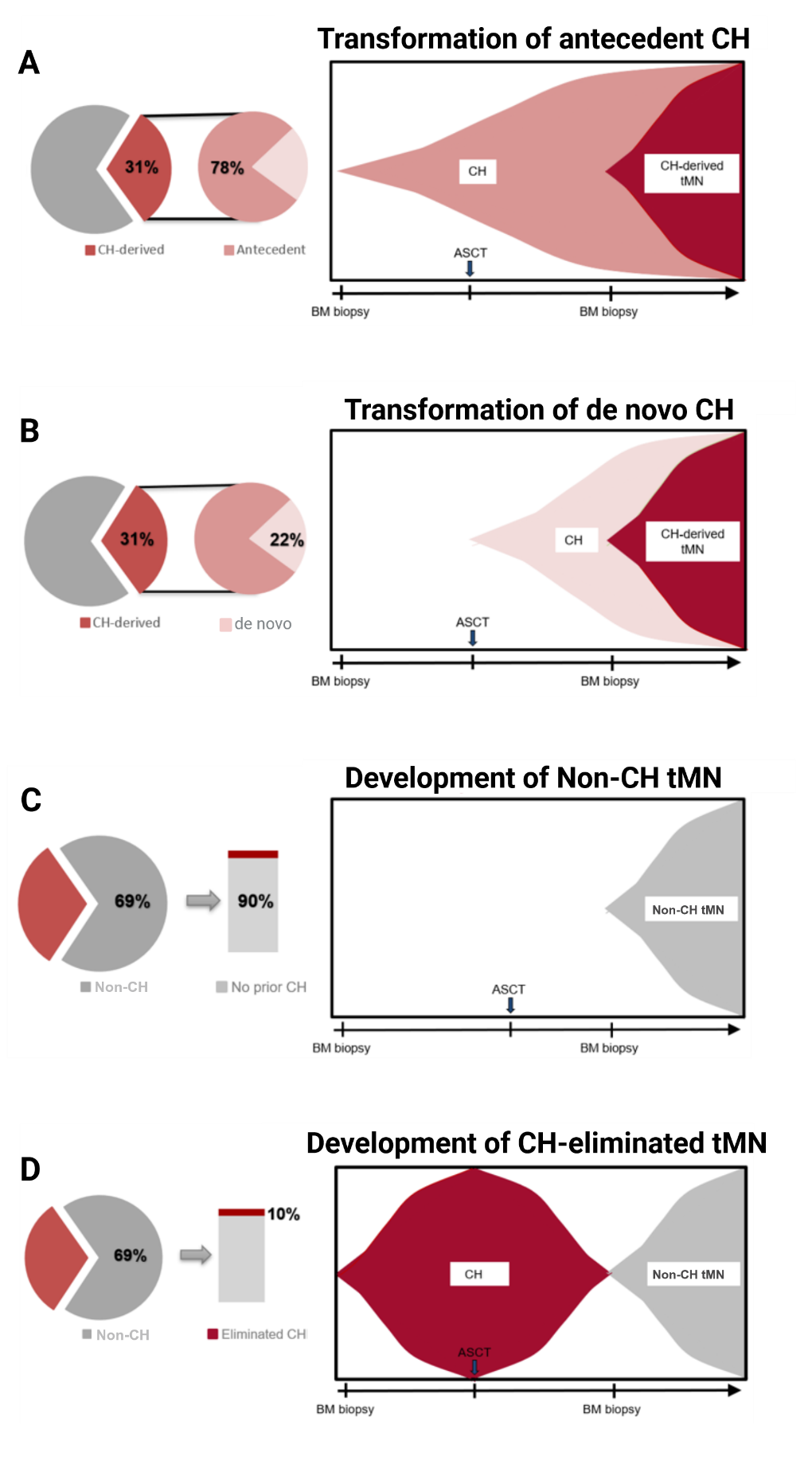
**

Schemes of the modes of post-aHSCT tMN development, including (A) the transformation of antecedent pre-aHSCT CH clones, (B) the transformation of post-aHSCT de novo CH clones, (C) the development of non-CH tMN that may also follow (D) the elimination of an antecedent CH clones.

**Supplementary Figure S11. Schemes and frequencies of modes of post-aHSCT tMN.**

**
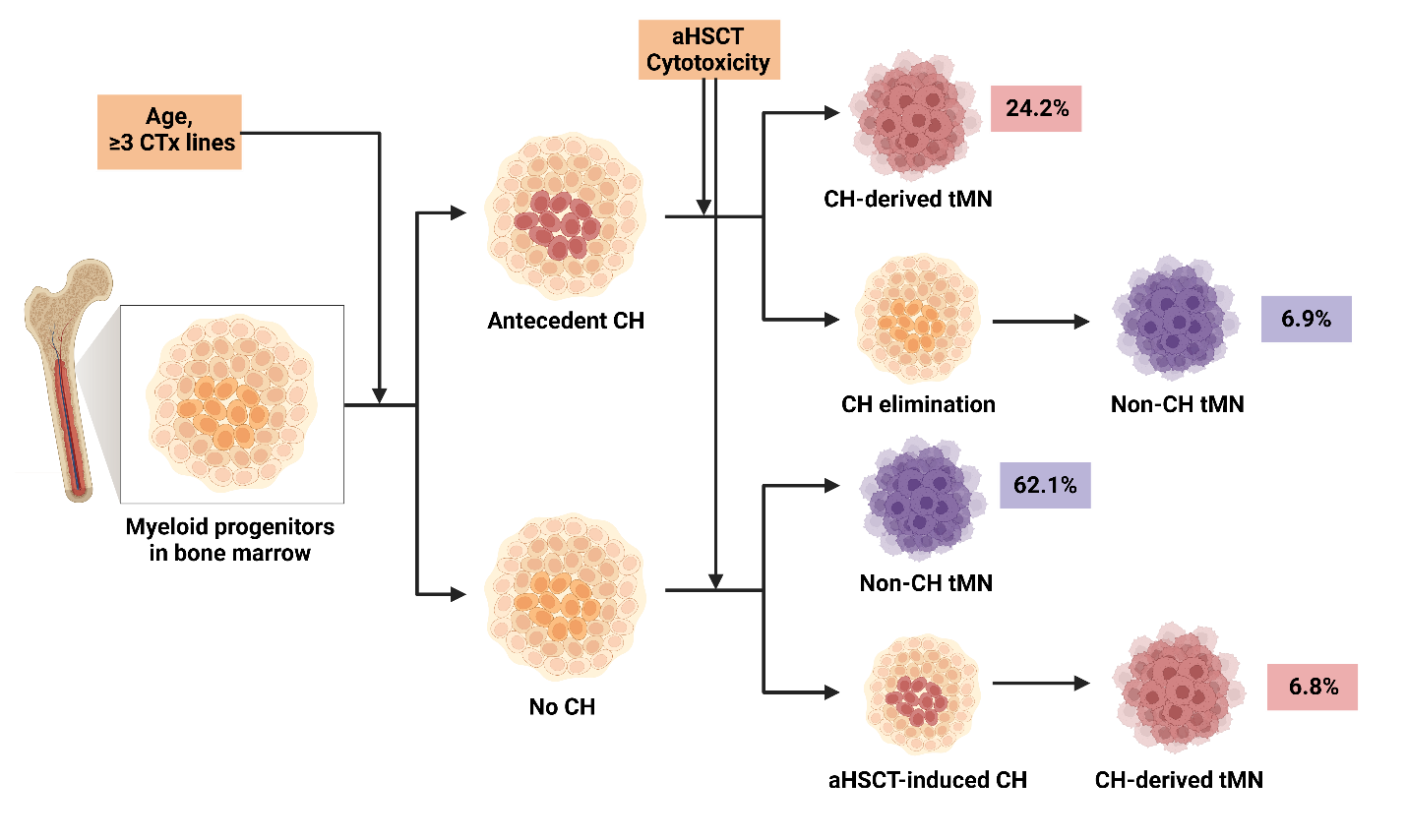
**

CTx : chemotherapy, CH : clonal hematopoiesis of indeterminate potential, aHSCT : autologous stem cell transplant, tMN : therapy-related myeloid neoplasm.

**References**

1. Coombs CC, Zehir A, Devlin SM, Kishtagari A, Syed A, Jonsson P*, et al.* Therapy-Related Clonal Hematopoiesis in Patients with Non-hematologic Cancers Is Common and Associated with Adverse Clinical Outcomes. *Cell Stem Cell* 2017 Sep 7; **21**(3)**:** 374-382.e374.

2. Guermouche H, Ravalet N, Gallay N, Deswarte C, Foucault A, Beaud J*, et al.* High prevalence of clonal hematopoiesis in the blood and bone marrow of healthy volunteers. *Blood Adv* 2020 Aug 11; **4**(15)**:** 3550-3557.

3. Abelson S, Collord G, Ng SWK, Weissbrod O, Mendelson Cohen N, Niemeyer E*, et al.* Prediction of acute myeloid leukaemia risk in healthy individuals. *Nature* 2018 Jul; **559**(7714)**:** 400-404.

4. Valent P. ICUS, IDUS, CHIP and CCUS: Diagnostic Criteria, Separation from MDS and Clinical Implications. *Pathobiology* 2019; **86**(1)**:** 30-38.

5. Khoury JD, Solary E, Abla O, Akkari Y, Alaggio R, Apperley JF*, et al.* The 5th edition of the World Health Organization Classification of Haematolymphoid Tumours: Myeloid and Histiocytic/Dendritic Neoplasms. *Leukemia* 2022 Jul; **36**(7)**:** 1703-1719.

6. Arber DA, Orazi A, Hasserjian RP, Borowitz MJ, Calvo KR, Kvasnicka HM*, et al.* International Consensus Classification of Myeloid Neoplasms and Acute Leukemias: integrating morphologic, clinical, and genomic data. *Blood* 2022 Sep 15; **140**(11)**:** 1200-1228.

7. Greenberg PL, Tuechler H, Schanz J, Sanz G, Garcia-Manero G, Solé F*, et al.* Revised international prognostic scoring system for myelodysplastic syndromes. *Blood* 2012 Sep 20; **120**(12)**:** 2454-2465.

8. Pfeilstöcker M, Tuechler H, Sanz G, Schanz J, Garcia-Manero G, Solé F*, et al.* Time-dependent changes in mortality and transformation risk in MDS. *Blood* 2016 Aug 18; **128**(7)**:** 902-910.

9. Steensma DP, Bejar R, Jaiswal S, Lindsley RC, Sekeres MA, Hasserjian RP*, et al.* Clonal hematopoiesis of indeterminate potential and its distinction from myelodysplastic syndromes. *Blood* 2015 Jul 2; **126**(1)**:** 9-16.

10. Xie M, Lu C, Wang J, McLellan MD, Johnson KJ, Wendl MC*, et al.* Age-related mutations associated with clonal hematopoietic expansion and malignancies. *Nat Med* 2014 Dec; **20**(12)**:** 1472-1478.

11. Jaiswal S, Fontanillas P, Flannick J, Manning A, Grauman PV, Mar BG*, et al.* Age-related clonal hematopoiesis associated with adverse outcomes. *N Engl J Med* 2014 Dec 25; **371**(26)**:** 2488-2498.

12. Genovese G, Kähler AK, Handsaker RE, Lindberg J, Rose SA, Bakhoum SF*, et al.* Clonal hematopoiesis and blood-cancer risk inferred from blood DNA sequence. *N Engl J Med* 2014 Dec 25; **371**(26)**:** 2477-2487.

13. Kwok B, Hall JM, Witte JS, Xu Y, Reddy P, Lin K*, et al.* MDS-associated somatic mutations and clonal hematopoiesis are common in idiopathic cytopenias of undetermined significance. *Blood* 2015 Nov 19; **126**(21)**:** 2355-2361.

14. Hirsch CM, Nazha A, Kneen K, Abazeed ME, Meggendorfer M, Przychodzen BP*, et al.* Consequences of mutant TET2 on clonality and subclonal hierarchy. *Leukemia* 2018 Aug; **32**(8)**:** 1751-1761.

15. Patel BJ, Przychodzen B, Thota S, Radivoyevitch T, Visconte V, Kuzmanovic T*, et al.* Genomic determinants of chronic myelomonocytic leukemia. *Leukemia* 2017 Dec; **31**(12)**:** 2815-2823.

16. Kuzmanovic T, Patel BJ, Sanikommu SR, Nagata Y, Awada H, Kerr CM*, et al.* Genomics of therapy-related myeloid neoplasms. *Haematologica* 2020 Mar; **105**(3)**:** e98-e101.

17. Heuser M, Thol F, Ganser A. Clonal Hematopoiesis of Indeterminate Potential. *Dtsch Arztebl Int* 2016 May 6; **113**(18)**:** 317-322.

18. Coleman JF, Theil KS, Tubbs RR, Cook JR. Diagnostic yield of bone marrow and peripheral blood FISH panel testing in clinically suspected myelodysplastic syndromes and/or acute myeloid leukemia: a prospective analysis of 433 cases. *Am J Clin Pathol* 2011 Jun; **135**(6)**:** 915-920.

19. Shaffer LG, Tommerup N. *ISCN 2005 : an international system for human cytogenetic nomenclature (2005) : recommendations of the International Standing Committee on Human Cytogenetic Nomenclature*. Karger: Basel ;, 2005.

20. Gonzalez Garcia JR, Meza-Espinoza JP. Use of the International System for Human Cytogenetic Nomenclature (ISCN). *Blood* 2006 Dec 1; **108**(12)**:** 3952-3953; author reply 3953.

21. Liehr T. International System for Human Cytogenetic or Cytogenomic Nomenclature (ISCN): Some Thoughts. *Cytogenet Genome Res* 2021; **161**(5)**:** 223-224.

22. Kaplan EL, Meier P. Nonparametric Estimation from Incomplete Observations. *J Am Stat Assoc* 1958; **53**(282)**:** 457-481.
